# Mechanochemical C–C bond stability as a function of substitution: a computational and experimental study

**DOI:** 10.1039/d6ra05049b

**Published:** 2026-07-29

**Authors:** Oleg Gouli, Hang Zhang, Elisa Ivry, Kanika Aggarwal, Charles E. Diesendruck

**Affiliations:** a Schulich Faculty of Chemistry, The Resnick Sustainability Centre for Catalysis Technion – Israel Institute of Technology Haifa 3200008 Israel charles@technion.ac.il

## Abstract

In polymer mechanochemistry, homolytic C–C bond scission is surprisingly ubiquitous, even in the presence of bonds that are more chemically or mechanically susceptible. Despite that, C–C bond scission is often viewed as a side reaction, although it can be harnessed for C–C bond activation and plastic recycling. Selective C–C bond scission reactions have been demonstrated mainly in strained or unusually long bonds in cyclic or very sterically crowded molecules. In this study, we employ the constrained geometries simulate external force (CoGEF) method to systematically explore how common substituents influence the mechanical stability of unstrained C–C bonds through electronic and steric effects. By identifying structural motifs that affect the calculated bond scission force, we provide insight into the mechanochemical stability, or potential mechanochemical recyclability of different polymer chemistries. Moreover, we introduce a new protocol to experimentally evaluate mechanochemical selectivity in mechanophores which do not provide clear spectroscopic signals, using poly(phthalaldehyde) mechanochemical depolymerization as a probe.

## Abstract 2

A combined computational and experimental strategy reveals how substituents control unstrained C–C mechanochemical reactivity.

## Graphical abstract

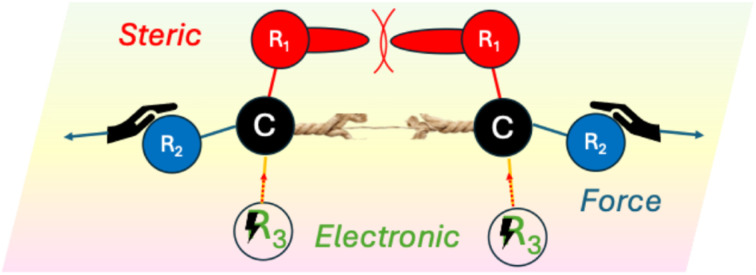

## Introduction

Chemical reactions driven or triggered by mechanical energy are the foundations of polymer mechanochemistry.^1^ Although grinding chemicals and materials is at the very birth of chemistry,^2^ research in mechanochemistry has boomed at the beginning of the 20th century. The mechanical grinding of plants and inorganic materials is well-documented historically, and yet, mechanical manipulations on organic molecules and polymeric materials towards target transformations are still developing topics.^3–5^ Covalent macromolecules in particular undergo interesting mechanochemical reaction pathways, as covalent bonds can be stretched to the point that the mechanical energy influences their entire potential energy surface,^6^ affecting chemical bonds' kinetics and thermodynamics, mostly accelerating covalent bond scission reactions. As a key example, in recent years, the application of mechanochemistry as an additional driving force for chemical recycling has gained significant momentum, either using mechanically susceptible functional groups, called mechanophores, or through direct homolysis of a polymer backbone.^7^ Indeed, the most ubiquitous polymer mechanochemistry reaction is the cleavage of simple and strong C–C bonds, which occur in carbon-based homopolymers such as polyolefins,^8^ as well as side-reactions in mechanophore-containing polymers.^9^ While the substituent effect on C–C bond-dissociation energy and other stability parameters are classical topics of study in thermal chemistry,^10,11^ few examples have looked at how carbon substituents affect the mechanochemical stability of the C–C bond. Such effects, are not only key parameters for identifying what plastics are more suitable for “mechanochemical” recycling, but also offer opportunities in accelerating C–C mechanochemistry, for example, in the context of C–C bond activation.^8^ Moreover, these effects complement previously explored structural strategies including modulation of the bond-force vector angle,^12–14^ exploiting “lever-arm” effects,^15–17^ and control of polymer morphology.^18–21^ In the past, the mechanical susceptibility of target C–C bonds has been enhanced through deliberate substitution patterns designed to create new mechanophores.^22,23^ Indeed, several mechanophores work through the homolytic scission of C–C bonds, but these systems primarily involve highly strained and/or elongated C–C bonds.^24–29^

Herein, we report a systematic computational survey of the mechanochemical scission of unstrained C–C bonds, examining how common substituents representing common monomer motifs in polyolefins influence susceptibility to mechanical force. To experimentally validate these findings, we synthesize a new “C–C mechanophore” containing a mechanically susceptible, but unstrained C–C bond, and evaluate its behaviour in solution using ultrasonication. Because this mechanophore does not produce a distinctive spectroscopic signal, we also describe a new method to evaluate scission selectivity by incorporating the C–C mechanophore into a polyphthalaldehyde (PPA) backbone.

## Results and discussion

### Computational modelling

**Fig. 1:**
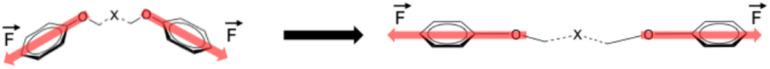
Molecular model structures used in CoGEF simulations. Phenoxy terminal groups are used as mechanically robust anchors, with X indicating the structural motif under investigation. Arrows indicate the simulated applied mechanical force.

In the “constrained geometries simulate external force” (CoGEF) method,^30,31^ molecular structures are minimized while the distance between two designated terminal atoms is incrementally increased to simulate mechanical strain over the chemical bonds thorough the vector connecting the end groups. In CoGEF, the direction of the applied load is defined by the vector connecting the two constrained terminal atoms at each step, thereby aligning the simulated deformation with the molecular axis expected to experience tension during mechanochemical treatment, as transmitted through the extension of the polymer segment. The data from these simulations is used to generate an energy-extension curve, with the bond rupture force (*F*_b_) determined from the slope near the bond-scission event (energy maximum). While this method does not take into consideration thermal effects or strain-rate effects,^6,32^ in general, the relative mechanical stability for bonds of a given series are reliably predicted.^31^ Indeed, CoGEF has been used to model multiple mechanophores and non-mechanophoric chemical bonds, showing good correlation with single-molecule force microscopy.^31^ To understand how small structural and electronic effects from substituents affect the force required to break unstrained C–C bonds, we carried out numerous CoGEF simulations of different simple unstrained carbon structures with different substituents around the target bond. The *in silico* modelling was carried out using Spartan with DFT at the B3LYP/6-31G* level of theory, under vacuum, following the typical parameters used to model mechanophores.^31^ The simple model-molecules tested were terminated on both sides by phenyl ether groups as mechanically robust anchors. Pulling at the *para* positions by gradually increasing the distance between the terminal atoms create longer force vectors which could better represent a chain pulling over pulling a terminal alkyl carbon, which could be more affected by bond and dihedral angles (Fig. 1). While Robb *et al*. have also carried out a large number of CoGEF studies on over 100 mechanophores using a different protocol,^31^ we decided to adapt to a different procedure as most molecules modelled here can be stronger than the end-caps used in the previously described protocol. The same CoGEF used for the C–C bonds was used to test the robustness of the C–O bond in PPA for comparison.^33^

Simulations were first done for simple alkanes (representing segments of polyethylene) to obtain baseline forces and standardize CoGEF parameters (such as end-cap effects). The initial calculations were done for different alkyl lengths (X = –(CH_2_)_5_– and up to –(CH_2_)_9_–), with varying distance increments (0.1 or 0.05 Å), and changing the number of data points used for the slope calculations (6, 8 or 12). The preliminary survey provided consistent C–C breaking force values independent of alkane length, with an average of 6.83 nN (standard deviation of 0.05 nN, for full details see the SI) and thus the parameters for our protocol were set to a step size of 0.1 Å and a slope of 6 data points. Also, as the experimental test for C–C bonds will be done using PPA, CoGEF was also carried out for a chain having two *o*PA repeating units, indicating a *F*_b_ of 5.46 ± 0.1 nN (Table 1, entry 1), establishing the limit value for mechanochemical selectivity in a PPA chain (which is described in the experimental part of the manuscript). Entry 2 presents the simple alkane X = –(CH_2_)_7_– as a reference which required a *F*_b_ = 6.76 ± 0.1 nN – *i.e.* the C–O bond in PPA is weaker than an unsubstituted C–C bond.^34–36^

**Table 1:** CoGEF calculations of forces required for C–C bond scission (*F*_b_) of model molecules

Entry	X	*F*_b_ a (nN)	Entry	X	*F*_b_ a (nN)
1	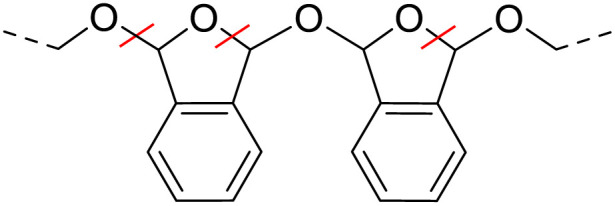	5.46 ± 0.06	19	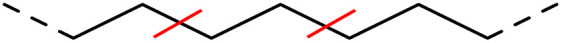	*trans* – 6.83 ± 0.03
2	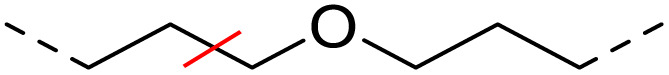	6.76 ± 0.01	20	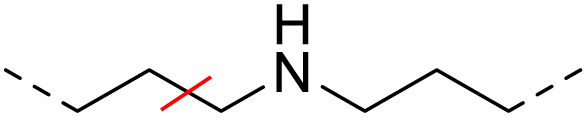	*cis* – 6.79 ± 0.03
3	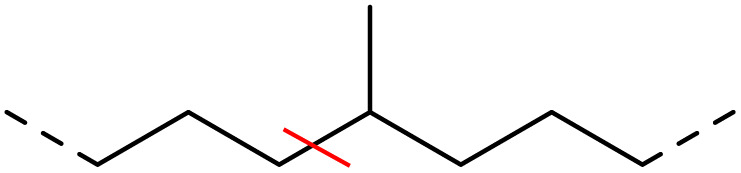	6.64 ± 0.07	21	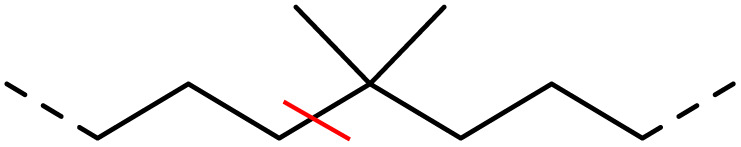	6.71 ± 0.04
4	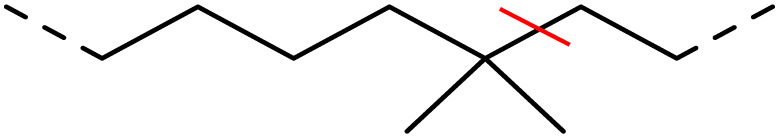	6.73 ± 0.03	22	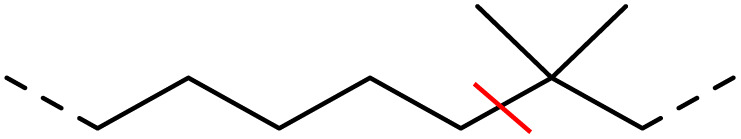	6.64 ± 0.03
5	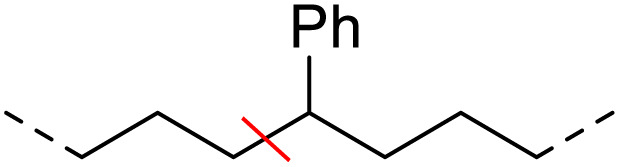	6.56 ± 0.03	23	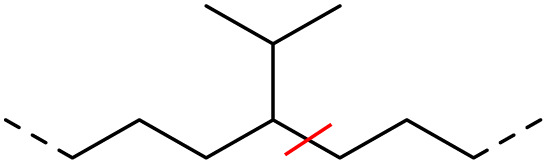	*trans* – 6.67 ± 0.02
			24		*cis* – 6.65 ± 0.02
6	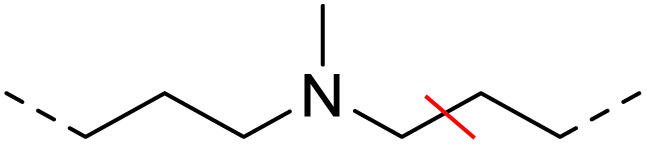	6.33 ± 0.03	25	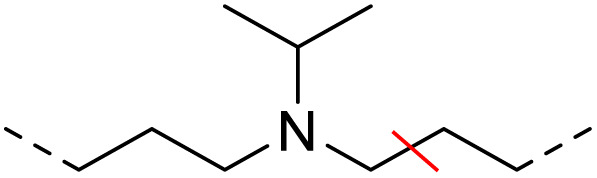	*trans* – 5.87 ± 0.04
			26		*cis* – 4.29 ± 0.04
7	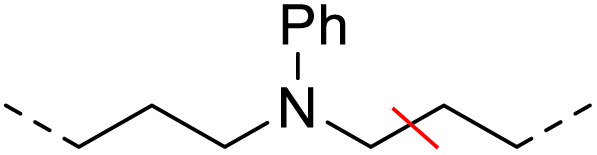	6.30 ± 0.03	27	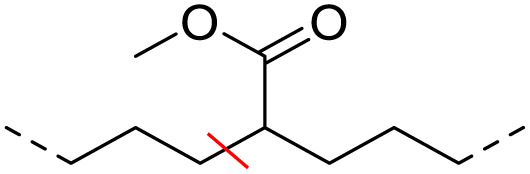	*trans* – 5.79 ± 0.02
			28		*cis* – 6.56 ± 0.02
8	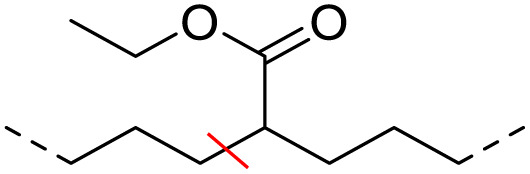	6.25 ± 0.04	29	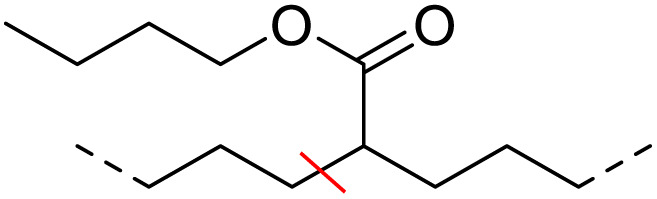	*trans* – 5.61 ± 0.04
			30		*cis* – 4.82 ± 0.05
9	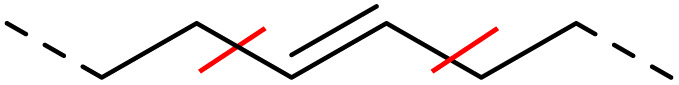	6.50 ± 0.01	31	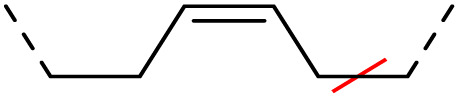	*trans* – 6.37 ± 0.02
			32		*cis* – 6.35 ± 0.03
10	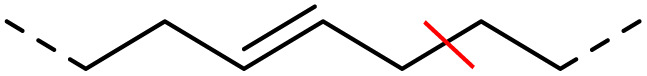	6.10 ± 0.03	33	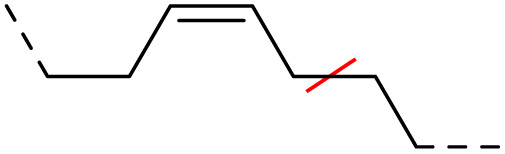	*trans* −6.49 ± 0.02
			34		*cis* – 6.43 ± 0.03
11	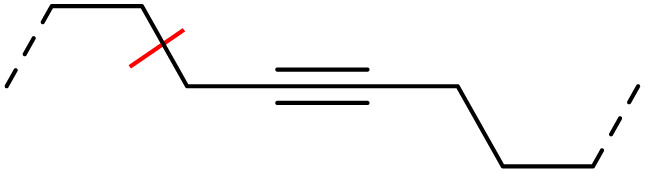	6.62 ± 0.03	35	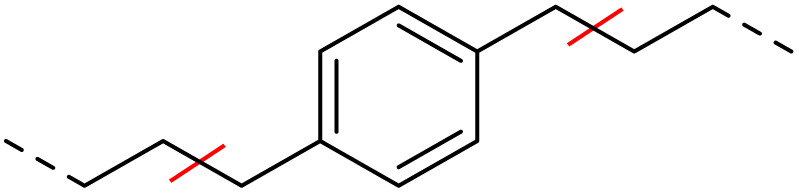	*trans* – 6.05 ± 0.03
			36		*cis* – 5.03 ± 0.05
12	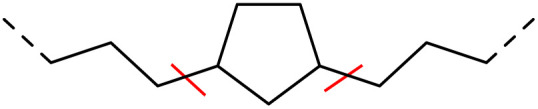	6.48 ± 0.03	37	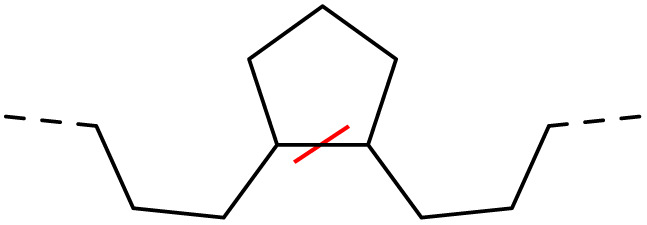	*trans* – 4.31 ± 0.04
			38		*cis* – 5.05 ± 0.4
13	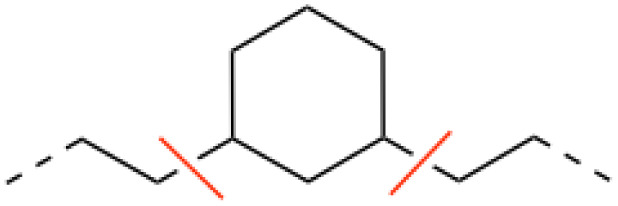	6.35 ± 0.04	39	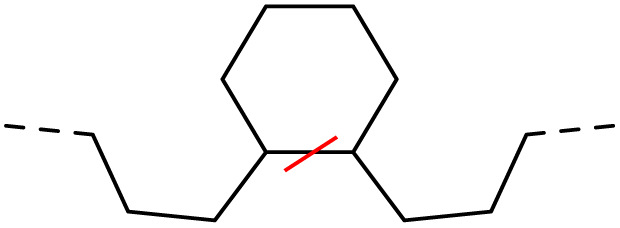	*trans* – 4.00 ± 0.03
			40		*cis* – 5.08 ± 0.05
14	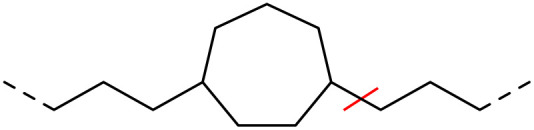	6.01 ± 0.04	41	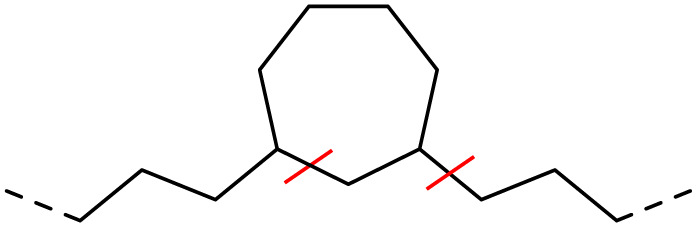	*trans* – 4.60 ± 0.04
			42		*cis* – 4.38 ± 0.05
15	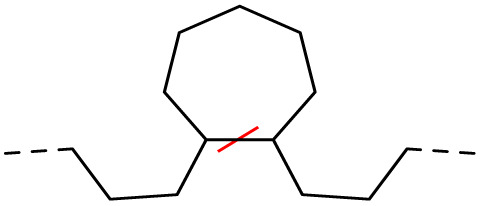	6.07 ± 0.05	43	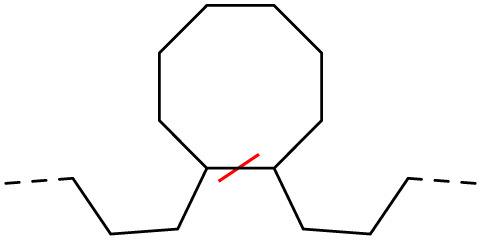	*trans* – 4.48 ± 0.03
			44		*cis* – 5.50 ± 0.06
16	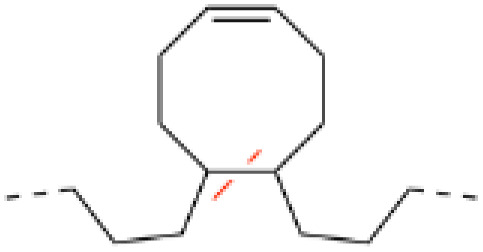	6.03 ± 0.04	45	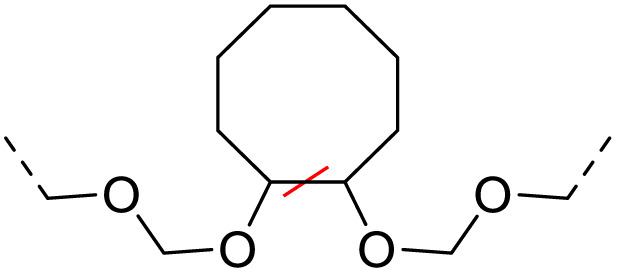	5.77 ± 0.03
17	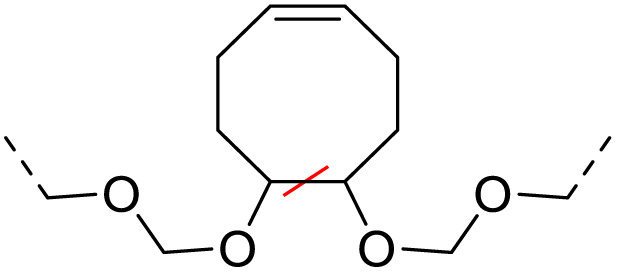	*trans* – 7.02 ± 0.03	46	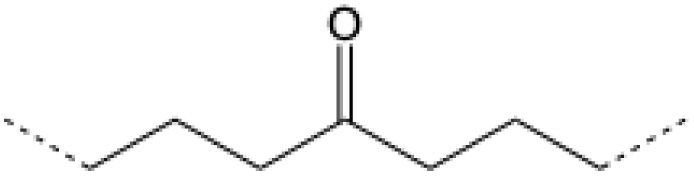	6.58 ± 0.03
18	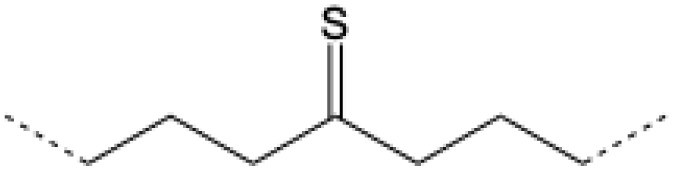	*cis* – 7.02 ± 0.03	47	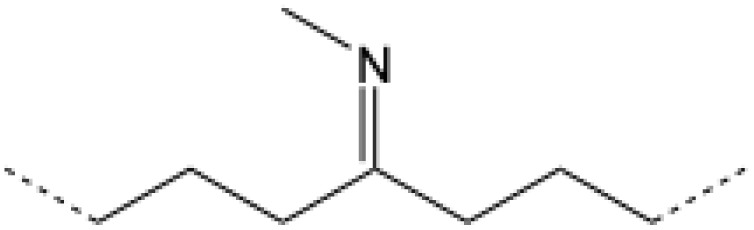	5.32 ± 0.05

a Calculated using Spartan 14 with DFT at the B3LYP/6-31G* level of theory, under vacuum, step size of 0.1 Å and 6 data points slope. The position of the calculated broken bond(s) is marked in red.

The model molecules were gradually changed to include alkane grafts, π-bonds, cyclic structures, and heteroatoms to create some polarization and/or radical stabilization or change the bond/force alignment. Over 40 structures bearing different structural centres were simulated and compared (Table 1, see the SI for detailed CoGEF plots). As a first electronic effect, the central carbon on this C7 structure was substituted by oxygen or nitrogen (representing a polyether or polyamine, entries 3 and 4), yet no significant change in the *F*_b_ was found, *i.e*., polarization of the backbone does not lower C–C bond mechanical stability – and the C-heteroatom does not become the weaker bond.^30^ Next, branches were added to the central carbon, which can stabilize the formed radical character during and after scission through both electronic and steric effects.^32^ Aliphatic structures with tertiary and quaternary carbons (representing polypropylene and polyisobutylene, entries 5 and 6) which, upon homolytic scission produce secondary and tertiary carbon radicals, lead to a more significant reduction in force by *ca.* 0.2 and 0.4 nN respectively. When the quaternary carbon is moved from the centre of the chain (entries 7 and 8), the *F*_b_ remains the same compared to a mid-chain position, yet the location of the scission follows the substituted carbon, emphasizing that this radical stabilizing effect reduces the mechanochemical stability of a polymer backbone and is, perhaps, capable of inducing some degree of regioselectivity. Surprisingly, using a phenyl ring instead of a simple methyl (providing stronger electronic effects) to produce a benzylic secondary radical (polystyrene, entry 9), did not provide an additional reduction in *F*_b_. Yet, increasing the branch volume (steric) with an isopropyl substituent (poly(3-methyl-1-butene), entry 10) reduced the *F*_b_ by additional 0.2 nN.

While the substitution of a central carbon by a –NH– had no effect on *F*_b_ (entry 4), if such nitrogen is tertiary, *F*_b_ is reduced with increasing the side-chain substituent volume (entries 11 and 12),^21,37^ even though we still observe a C–C scission leading to a primary aliphatic radical, at *F*_b_ similar to that of a secondary radical (entry 5). An *N*-phenyl group resulted in further electronic stabilization (entry 13), producing a primary radical at the same *F*_b_ as an aliphatic substituent producing a tertiary radical (entry 6). As such, more significant effects were found by using ester groups as electron withdrawing groups (acrylic polymers, entries 14–16), which reduced the *F*_b_ by 0.8 nN. These results suggest that acrylic/methacrylic polymers are more prone to mechanochemical C–C bond scission compared to polyolefins. Interestingly, these two classes of polymers (polyethylene-*co*-propylene and polymethacrylates) are used as viscosity modifiers in the lubricant industry, and their solution shear stability is one of the important parameters defining the service life of the oil.^38^ Next, the presence of double and triple bonds was investigated, which could act as lever-arms while also having an electronic allylic effect on the radical stability.^17^ Unexpectedly, the presence of such bonds strengthened the single C–C bonds, with CoGEF showing slightly higher *F*_b_ values (polybutadiene, polyynes, entries 17–21). In addition to these linear structures, the bank of model compounds was expanded to molecules containing cyclic structures, but limited to those with little ring strain (5–8 membered rings, cyclic olefin polymers).^39^ These compounds can undergo a mechanochemical exocyclic bond scission (lever effect)^15^ or a ring-opening, which could be followed by a secondary bond scission.

For model structures showing exocyclic C–C bond scission (entries 23, 24, 27, 28 and 31–34), CoGEF did not indicate a lever-arm effect – *i.e.*, no significant reduction in *F*_b_ was found in the simulation. However, when the simulated puling led to a ring-opening reaction, the required *F*_b_ was consistently lower (entries 25, 26, 29, 30 and 35–44). Interestingly, the deciding factor between a high *F*_b_ exocyclic and lower *F*_b_ ring-opening mechanochemical reaction is the distance between the polymer attachment points to the cyclic structure. When the polymer is connected through neighbouring carbons, a ring-opening reaction was observed. If the polymer is connected through distal 1,3 or 1,4 positions, exocyclic C–C scission was preferred. It has been shown that C–C bonds in strained rings such as *gem*-dichlorocycloproprane (*F*_b_ = 3.2–3.8 nN) and benzocyclobutene (*F*_b_ = 3.1–4.1 nN) undergo scission more easily.^31^ Yet, in our studies, rings with even minimal strain (compared to cyclobutane with ring strain of 26.5 kcal mol^−1^),^39^ such as cyclopentane (6.2 kcal mol^−1^, entries 23–26), cycloheptane (6.3 kcal mol^−1^, entries 31–34), cyclooctane (9.7 kcal mol^−1^, entries 35–44), and even cyclohexane with 0.0 kcal mol^−1^ as reference point (entries 29–30) also showed lower activation forces, with *cis*-1,2-cyclopentane (entry 26), *trans*-1,2-cyclooctane (entry 37), and *trans*-1,2-cyclooctene (entry 39) showing the most promising results, placing these structures in the typical *F*_b_ range of mechanophores.^31^

The eight-membered rings were re-run with ether connectors, given that the connector can electronically affect the target bond for scission.^40^ For *trans*-cyclooctane-1,2-diol, introduction of a short C–O backbone (polyformaldehyde) slightly increased the *F*_b_ (entries 41 compared to entries 37). Introducing a secondary degree of strain to the ring in the form of endocyclic double bond using a *trans*-5-cycloocetene-1,2-diol brought down the *F*_b_ to 4.48 nN (entry 43). Finally, carbonyl substituents were also added (entries 45–47) to verify their effects. Ketone introduction (polyketone) has a quite strong effect, reducing *F*_b_ by 1.2 nN. This effect disappears in thioketone, indicating this is an electronic effect. Interestingly, if the carbonyl is converted to an imine, the effect is even stronger, further reducing *F*_b_.

**Fig. 2:**
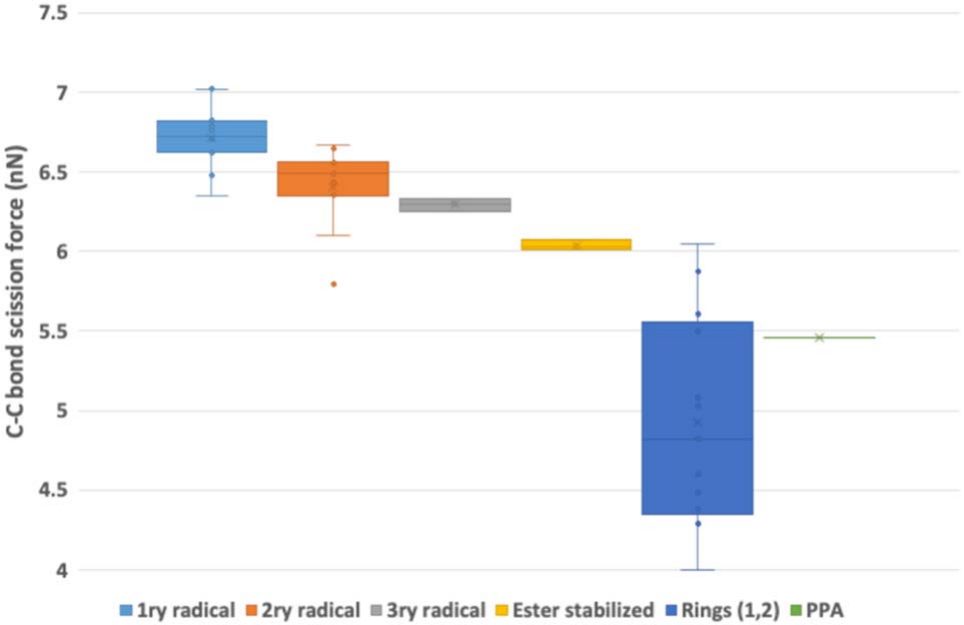
Summary of CoGEF-calculated maximum bond scission forces (*F*_b_) for different substitution patterns. Values are groups by radical stabilization class: disubstituted C–C which form primary radicals (Table 1 entries 2–4, 11–13 and 17–22); trisubstituted, forming secondary radicals, including rings with distal attachments (entries 5, 9–10, 23, 24, 27, 28 and 31–34); tetrasubstituted breaking into tertiary radicals (entries 6–8); ester-stabilized C–C forming secondary radicals (entries 14–16) and cyclic ring-opening structures connected through 1,2 attachments (entries 25, 26, 29, 30 and 35–44). All compared to PPA (entry 1).

To summarize the CoGEF simulation results, it seems like radical stabilization is a key effect in increasing C–C mechanical susceptibility. This can be clearly seen when grouping structures by the type of radical stabilization (Fig. 2). Yet, C–C bonds in cyclic molecules, particular connected to the polymer chains in an anti-attachment, still leads to the most significant reduction in *F*_b_.^29^

### Experimental validation

PPA, arguably the most studied polyacetal (backbone exclusively made of C–O bonds),^41^ is made by polymerization of *o*-phthalaldehyde (*o*PA), producing linear or cyclic chains depending on the initiator.^42,43^ PPA needs to be end-capped due to its low ceiling temperature (*T*_c_ = −43 °C), providing a solid polymer which is kinetically trapped at room temperature. When PPA chains are mechanically stressed, mechanochemical C–O bond scission occurs heterolytically, producing charged chain-ends (uncapped) which, at room temperature, rapidly unzip back to the *o*PA monomer.^6,44^

Therefore, if a PPA has, for example, a mechanophore at the chain centre, upon mechanical stress, mechanophore activation might occur instead of C–O heterolytic scission, which does not lead to PPA depolymerization. Nevertheless, some non-selective reactions still occur, leading to unzipping. By comparing the rate of PPA depolymerization, one can estimate the selectivity of the mechanophore, particularly taking into consideration that there is a single mechanophore for tens of *o*PA mononomers at the high stress chain centre.

**Scheme 1:**
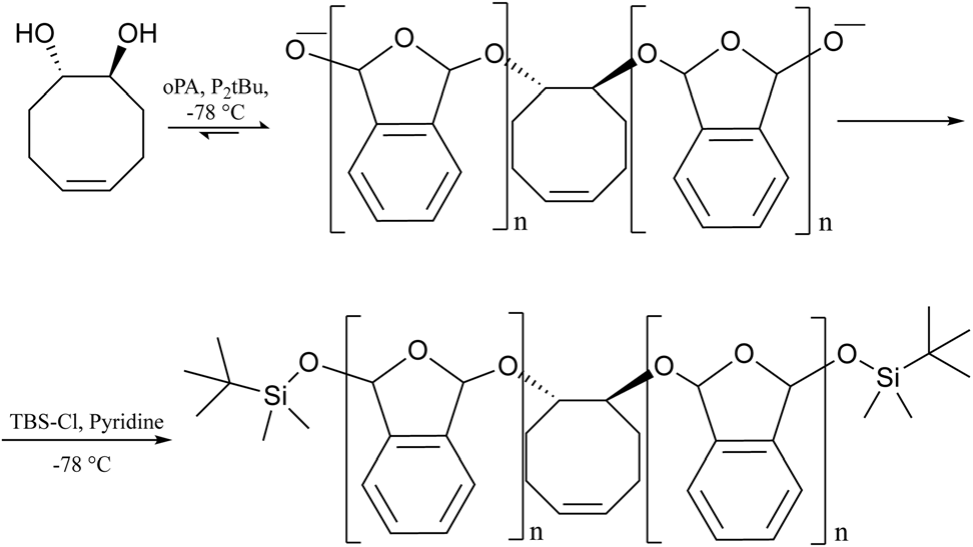
Synthesis of *Z*-5-cyclooctene-1,2-*trans*-diol centred PPA. Reactions conditions: polymerization obtained for 1.0 eq. of *o*PA monomer (7.45 mmol) and catalytic amount of phosphazane base (P_2_*t*Bu, 0.054 mmol).

To validate the simulation results, we chose *Z*-5-cyclooctene-*trans*-1,2-diol as a potential unstrained C–C mechanophore. The diol can be used as a di-initiator in the anionic polymerization of *o*PA, incorporating the eight-membered ring at the centre of a PPA chain. *Z*-5-Cyclooctene-*trans*-1,2-diol was prepared by mono-epoxidation of 1,5-cyclooctadiene followed by acid catalysed water addition, to obtain the *trans*-1,2-diol (Scheme 1).^45^ To achieve high enough molecular weights (*M*_w_), the *o*PA monomer was recrystallized trice and then dissolved in anhydrous THF in a glove-box, before addition of the initiator, which was dehydrated over molecular sieves.^44^ The flask was sealed, removed from the glove-box, cooled to −78 °C under N_2_ pressure before injection of the phosphazene base 1-*tert*-butyl-2,2,4,4,4-pentakis(dimethylamino)-2Λ5,4Λ5-catenadi (phosphazene) (P_2_-*t*-Bu) in a THF solution (Scheme 1). After mixing for 4 h at −78 °C, pyridine and *tert*-butyldimethylsilyl chloride (TBS-Cl) in anhydrous THF were injected for capping of the polymer. After stirring for additional 3 h at −78 °C, the solution was allowed to return to room temperature. The resulting polymer (PPA-a) was then precipitated in diethyl ether, filtered, dried under vacuum for 48 h, and stored under argon at −18 °C. Using the same polymerization protocol, 1,7-heptanediol was also used as a di-initiator providing a PPA with a –(CH_2_)_7_– aliphatic centre as a C–C baseline control (PPA-b). 1-Heptanol was also used as initiator for a second control in which the C–C bonds are located at the chain end of the polymer (PPA-c).

**Fig. 3:**
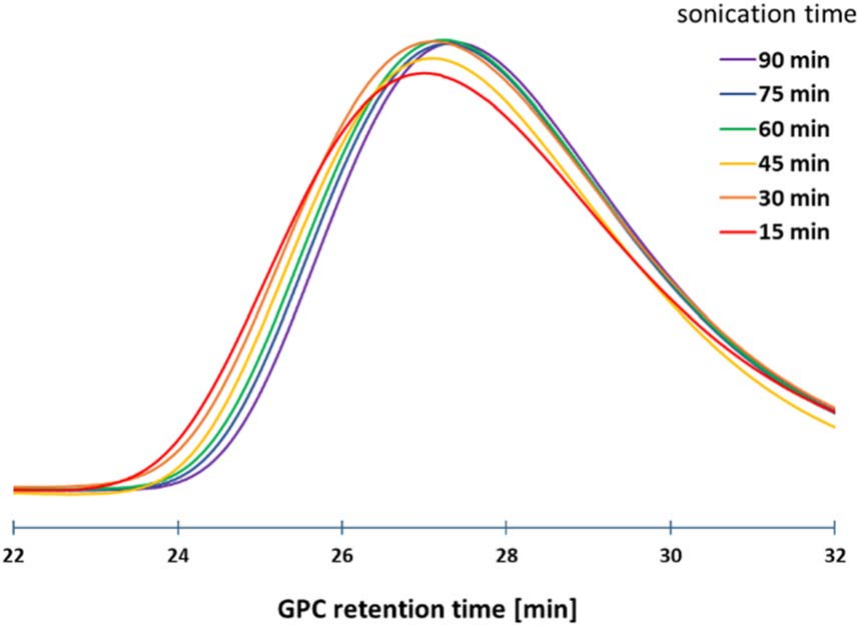
SEC analysis (dRI signal) of PPA-a under ultrasonication (4 mg mL^−1^ in dry THF, Suslick cell, −8 ± 2 °C) over time, showing a small change in peak retention time.

**Fig. 4:**
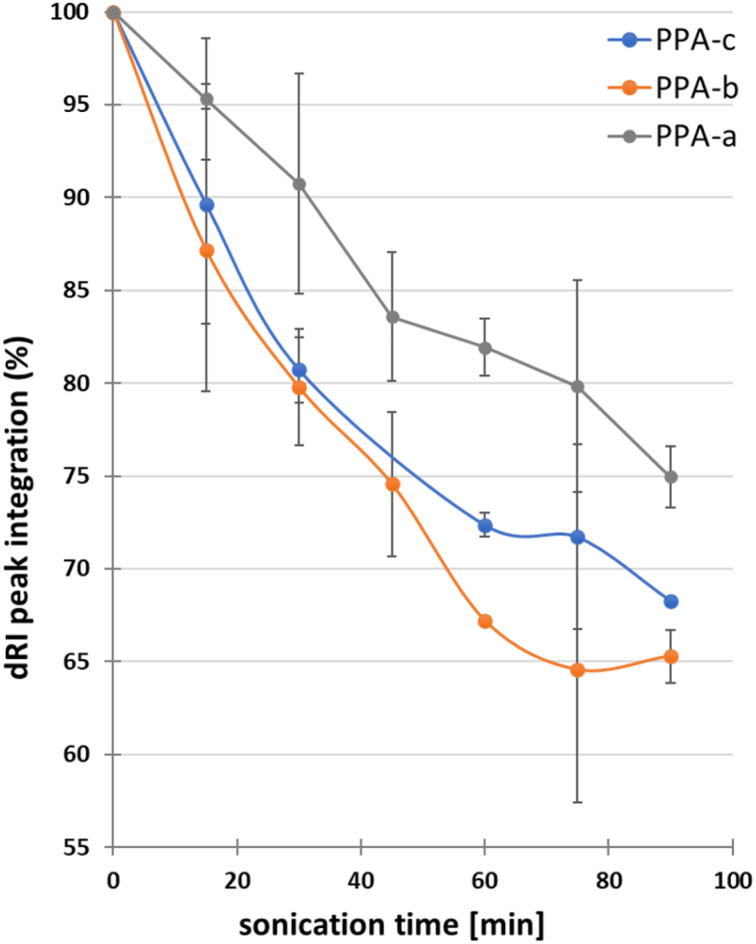
Decay in dRI peak area for PPA-a–c during sonication (4 mg mL^−1^ in dry THF, Suslick cell, −8 ± 2 °C), representing mechanochemically induced depolymerization of the PPAs. Error bars represent standard deviations from three independent experiments.

The three PPAs were characterized by seize-exclusion chromatography (SEC), presenting very similar *M*_w_s and dispersities (*Đ*, Table 2 and Fig. S1, SI). The polymers were then mechanically stressed in solution using ultrasonication induced cavitation.^46^ Given the differential refractive index (dRI) detector was used to quantify the reaction rate, the sonication was done at relatively high concentration (4 mg mL^−1^ in dry THF in Suslick cell) to have higher signal and accuracy; and, most importantly, above the ceiling temperature of PPA (at −8 ± 2 °C).^44^ The sonication experiments (pulsing 1 s on, 2 s off) were done in triplicates for 1.5 h with active cooling during which samples were taken every 15 min and analysed by SEC, while integrating the polymer peak in the dRI detector. Given the dRI signal is proportional to polymer concentration, and all samples are directly injected (without any significant changes in overall monomer concentration), reduction in the polymer peak area indicates polymer depolymerization, which is accompanied by an increase in the low *M*_w_ peak due to the formation of *o*PA monomers.^44^ As an example, Fig. 3 presents the SEC results of PPA-a over sonication time. Comparison of the decrease in polymer peak area for all three PPAs (Fig. 4), shows similar, quite rapid fall in polymer area (concentration) for control PPA-b and PPA-c, reaching similar degree of depolymerization after 90 min. PPA-c, having no C–C bonds at the chain centre, provides a baseline of the depolymerization of the PPA in which only C–O bond mechanochemistry can occur and thus every mechanochemical reaction leads to thermal chain depolymerization. PPA-b, has a few C–C bonds at the chain centre, yet, with a *F*_b_ of 6.76 nN (above described CoGEF), which is stronger bond than the multiple C–O bonds of PPA (with calculated *F*_b_ of 5.46 nN). Therefore, it is expected that statistically abundant C–O bonds around the chain centre will present significantly more scission events than the C–C bond, leading to depolymerization very similar to PPA-c. Finally, PPA-a, which includes a mechanically weaker C–C bond relative to the C–O backbone at the chain centre (PPA-b, *F*_b_ = 5.46 nN), presents a significantly inhibited decay in polymer peak area (although some PPA depolymerization is still seen). This indicates that while the scission of this C–C bond is not completely selective in ultrasonication, considering the polymer has a single “weak” C–C bond amongst tens of C–O bonds at the same overstretched segments, a significant enhancement in C–C bond scission selectivity is demonstrated. A Student's *t*-test confirms that the behaviour of PPA-a is statistically different from PPA-b and PPA-c (see Table S1).

**Table 2:** Parameters of synthesised PPAs from different initiators

Entry	Initiator^a^	*M*_w_ [kDa]	*M*_n_ [kDa]	*Đ*
PPA-a	*Z*-5-Cycloocten-*trans*-1,2-diol	51.2	36.9	1.39
PPA-b	1,7-Heptanediol	56.8	40.0	1.42
PPA-c	1-Heptanol	56.5	40.8	1.38

a 0.09 mol% relative to the *o*PA monomer used in polymerization.

## Conclusions

The CoGEF method was used to simulate how different substitution patterns on the carbons of C–C bonds affect the required force for mechanochemical C–C bond scission. Dozens of structures were modelled and the most significant reduction in scission force was found for cyclic structures, even with minimal ring strain, although other substitutions also present significant effects relative to –CH_2_–CH_2_–, particularly substituents that stabilize radical carbons either electronically or sterically. Indeed, addition of an ester group (equivalent to an acrylate) significantly reduced the force required for C–C bond scission, therefore indicating that, at the chemical level, acrylic plastics are more prone to mechanically accelerated chemical recycling compared to say polyethylene (in actual solid-state recycling other parameters such as glass transition temperature and entanglements would also play a role).^47^ Yet, given the cyclic structures showed the most significant result, *Z*-5-cyclooctene-1,2-*trans*-diol was synthesized and used as a di-initiator to produce a chain-centred simple C–C “mechanophore” in a PPA chain. Control PPAs were prepared using 1,7-heptanediol, which installs stronger, acyclic unsubstituted C–C bonds at the chain centre; and 1-heptanol, which has only C–O bonds at the chain centre. Under mechanochemical stress induced by sonication, the two control PPA polymers, depolymerized at similar rates, indicating that mechanochemical scission was occurring at C–O bonds. Meanwhile, the PPA with the chain-centred cyclooctene mechanophore showed a significantly reduced rate of depolymerization, supporting a significant amount of C–C bond scission, even if there are few C–C bonds compared to C–O in the polymer overstretched segments. This indicates that cyclooctenes can act as a relatively unstrained selective C–C mechanophore within a C–O backbone. This study offers mechanistic insights that could guide the rational design of regioselective mechanophores, as well as provide information on the overall mechanochemical stability of different monomers – whether in structural materials or in applications that exploit mechanical stress to accelerate recycling. In addition, a new strategy and protocol for testing mechanophore selectivity based on PPA depolymerization was described. Unexpectedly, PPA showed relatively low activation force (5.5 nN) for a C–O based polymer, limiting the C–C bonds that can be tested, which need to be quite active, but such methodology can be useful in testing mechanophores that are hard to characterize spectroscopically.

## Conflicts of interest

There are no conflicts to declare.

## Supplementary Material

RA-OLF-D6RA05049B-s001

## Data Availability

The data supporting this article are included as part of the supplementary information (SI). Supplementary information: description of computational modelling plots, synthetic procedures, NMR characterization, SEC and statistical analysis. See DOI: https://doi.org/10.1039/d6ra05049b.
