# Supplementary material for: Mechanochemical C–C bond stability as a function of substitution: a computational and experimental study
Source: RSC Adv. 2026 Jul 29. Online ahead of print. doi: 10.1039/d6ra05049b (PMC13417707; doi:10.1039/d6ra05049b)
Supplement: RA-OLF-D6RA05049B-s001 [file RA-OLF-D6RA05049B-s001.pdf]

Supplementary Information (SI) for RSC Advances.
This journal is © The Royal Society of Chemistry 2026

Supplementary Information

Mechanochemical C–C Bond Stability as a Function of Substitution: A Computational and Experimental Study

Oleg Gouli,[a] Hang Zhang,[a] Elisa Ivry,[a] Kanika Aggarwal[a] and Charles E. Diesendruck*[a]

[a] Schulich Faculty of Chemistry and the Resnick Sustainability Center for Catalysis Technion − Israel Institute of Technology, Haifa 3200008, Israel

Table of Content

Part I. General Information **3**
Part II. CoGEF Modeling **4**
General Method 4
Determination of F_b 4
CoGEF calculations 5
Part III. Synthetic Procedures **22**
Part IV. NMR Spectra **23**
Part V. GPC Analysis **24**
Part VI. Statistical Analysis **28**

Part I. General Information

All materials, unless otherwise stated, were purchased from commercial sources, and used without further purification. All solvents were dried prior to use. Purification by column chromatography was performed on Davisil grade chromatographic silica media 60 Å (35-75 μm, 220-440 mesh). TLC analyses were performed using Merck precoated silica gel (0.2mm) aluminum (backed) sheets. NMR spectra were recorded using an AVANCE III 400 MHz Bruker and AVANCE III 300 MHz Bruker spectrometers. Chemical shifts (δ) are reported in ppm relative to residual $CHCl_3$ as internal reference. GPC analyses were performed using a Thermo LC system equipped with TSK gel Guard Column HHR-L and 4 TSK gel G4000HHR columns in sequence. Detection was obtained with a penta-detector system including Dionex DAD-3000 PDI UV-Vis Detector, Wyatt Viscostar II, Wyatt OPTILAB T-rEX, Wyatt MALS DAWN HELEOS II 8+TR and Wyatt QELS DLS. GPC data analysis was done using Wyatt's Astra 6 software.Ultrasonication was done in home-made Suslick cells using a SONICS Vibra-cell ultrasonic processor 500 Watt at 9.57 W/cm^2 power intensity, 20 kHz frequency, and pulsing 1 sec 'on' 2 sec 'off'.

Part II. CoGEF Modeling

General Method

Modeling was carried out with Spartan 14 V1.1.8 or Spartan 18 V1.4.4, using DFT at the B3LYP/6-31G* level of theory, in vacuum. For each molecule, the equilibrium geometry and its energy were initially calculated via the minimization function. Different target "X" alkyl chains (see Figure S1) were modelled when attached via an ether bond to terminal phenyl rings, as these are more mechanically strong than the target C-C bonds. All the X targets studied are long enough that the electronic effect of the O atom is ignored - as shown below, when an ether group is included in X. The “pulling” force was simulated by gradually increasing the distance between the designated carbon atoms at the edges of the model compound (carbons para to the O atom), as described in figure S1. At each step, the distance was increased, the geometry minimized, and its energy calculated. At a certain distance, it is energetically favorable for a covalent bond to break and allow other bonds to relax to typical bond lengths, and therefore, the energy of the system is significantly reduced after releasing the accumulated strain. These energies are plotted and the slope of the graph just prior to the breaking point is the maximal force required for the bond scission (F_b). This was calculated by using a linear regression for the last data points of the graph and computing the slope (derivative of energy over distance), using Microsoft Excel data analysis algorithm. The computed value of the slope is then divided by the Avogadro number to provide the final values in nN units.

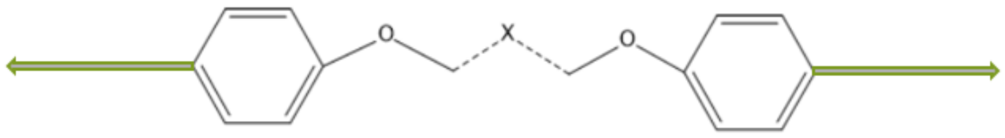

Figure S1. Repeatable anchor units structure used in CoGEF method, arrows indicate simulated puling force

Method Consistency

To verify if the model compound and different parameters taken are adequate, we initially modelled short linear alkane chains -$(CH_2)_x$- while changing: software (SW) version, step size (0.1 or 0.05 Å), number of slope data points (4, 6, 8, 12) and number of carbons chain (5, 7, 9), as presented in Table S1. The results in this first part indicate that the CoGEF protocol in Spartan provides consistent and reliable results, with the highest difference in force being less than 0.1 nN (~1.2%). Further modeling was set to step size of 0.1 Å, a slope of 6 data points using Spartan 14.

Table S1. Different parameters varied to test CoGEF calculations using C-C bond scission in short alkane chains.

Step size (Å)	SW version	# of points	Chain length	F_b (nN)
0.05	14	12	7	6.78
0.05	14	6	7	6.85
0.1	14	6	7	6.76
0.1	18	6	7	6.88
0.1	18	4	7	6.92
0.1	18	8	7	6.83
0.1	18	6	5	6.85
0.1	18	6	9	6.79
		Average		6.83
		Standard deviation		0.05

<u>CoGEF calculations of forces required for C-C bond scission (F_b) of all model molecules</u>

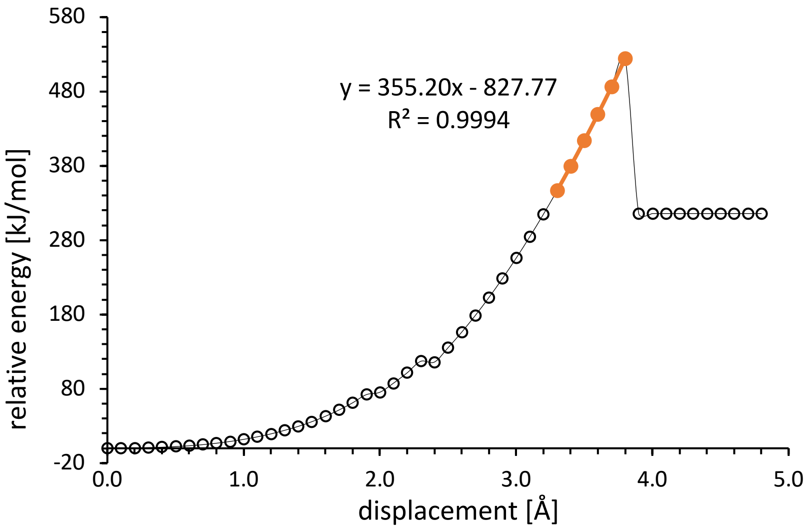

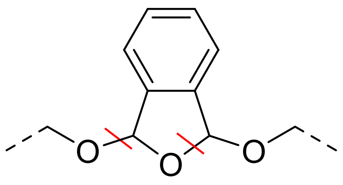

1'

F_b = 5.90±0.07

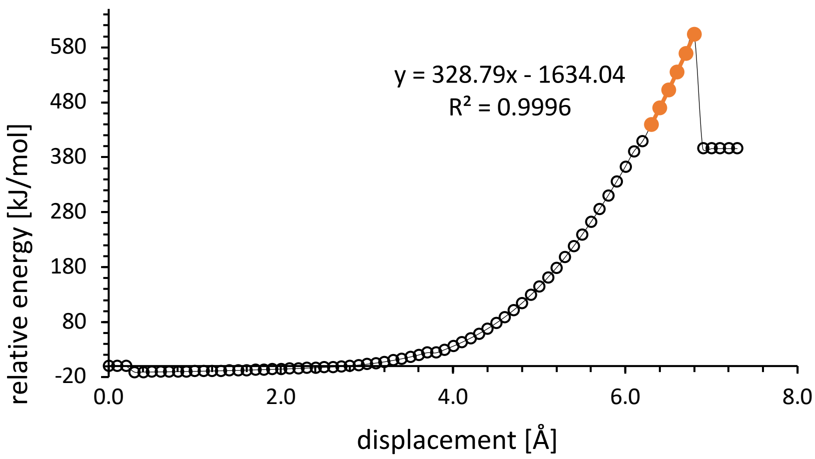

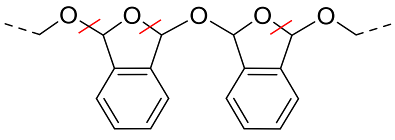

1

F_b = 5.46±0.06

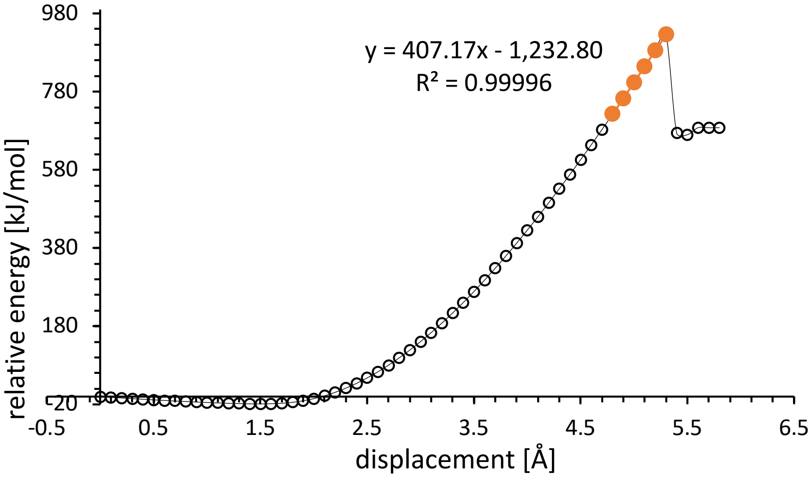

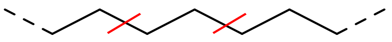

2

F_b = 6.76±0.01

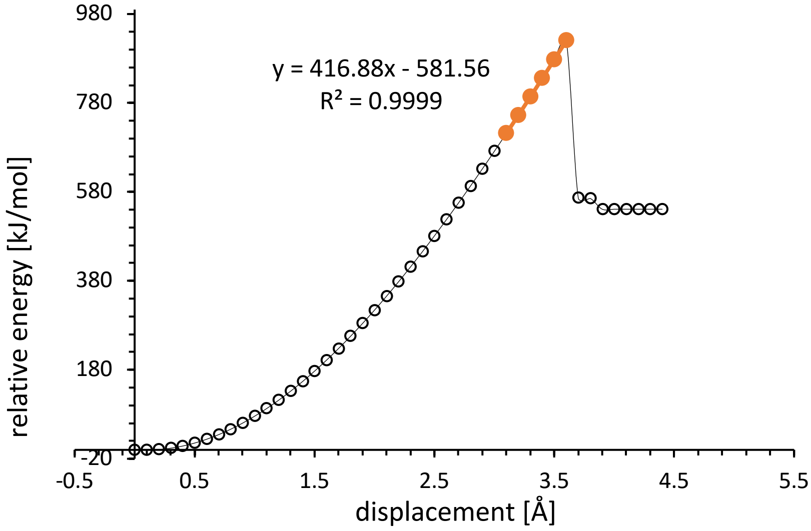

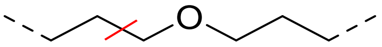

3

F_b = 6.64±0.07

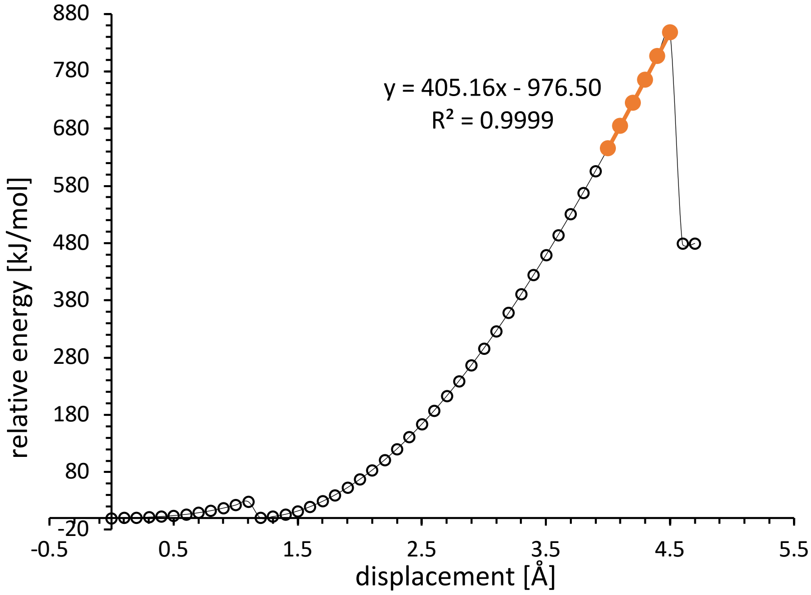

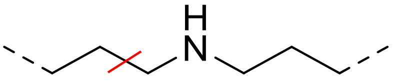

4

F_b = 6.73±0.03

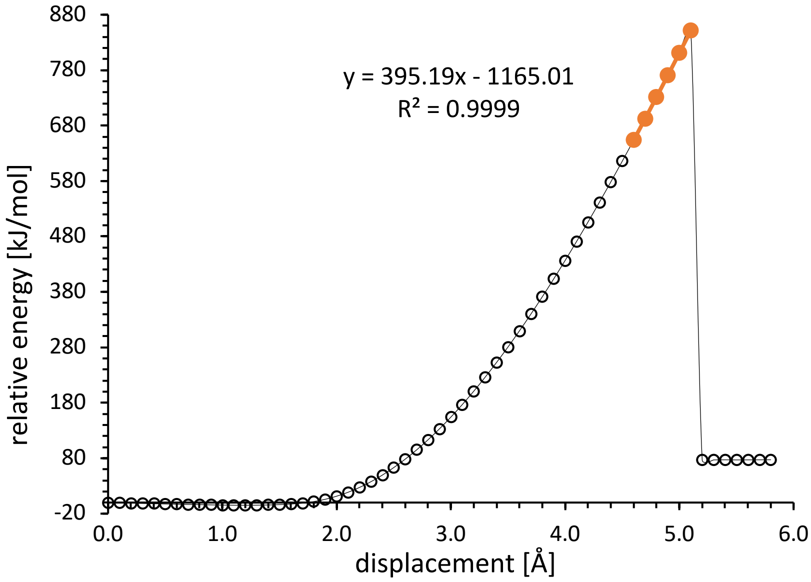

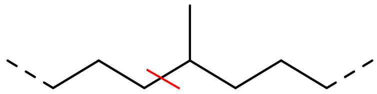

5

F_b = 6.56±0.03

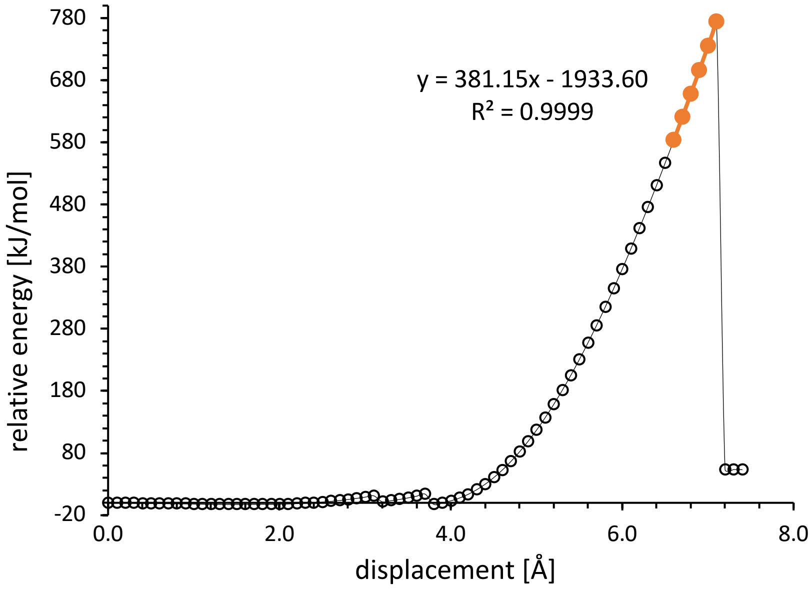

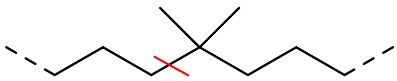

6

F_b = 6.33±0.03

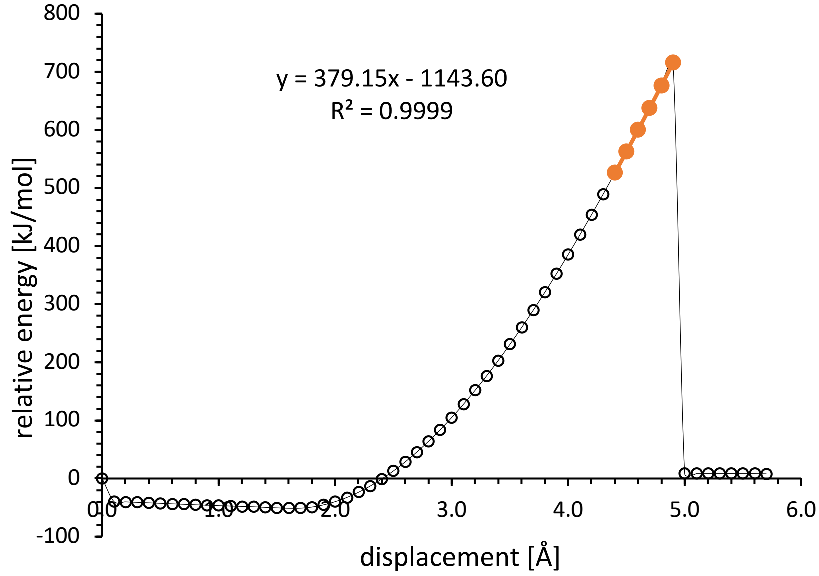

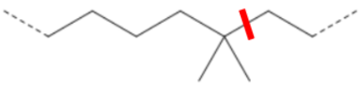

7

F_b = 6.30±0.03

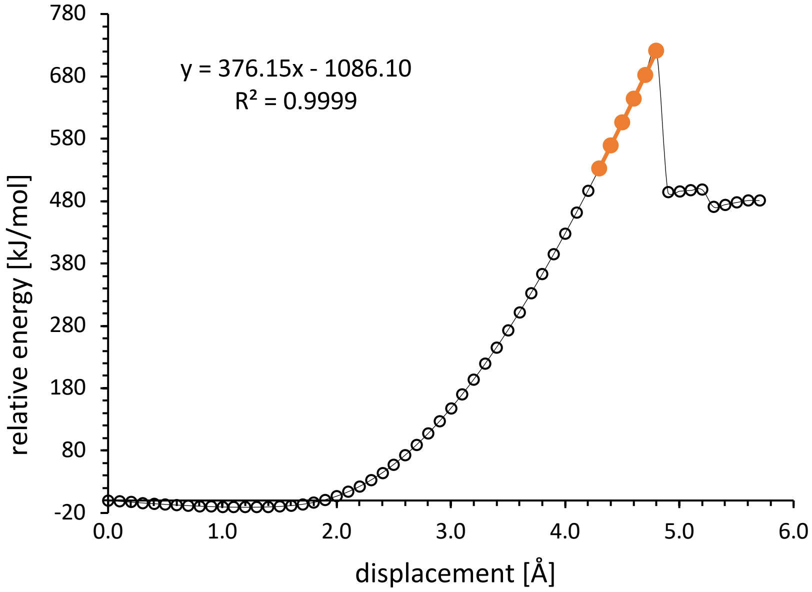	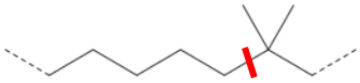<br>**8**<br>F_b = 6.25±0.04
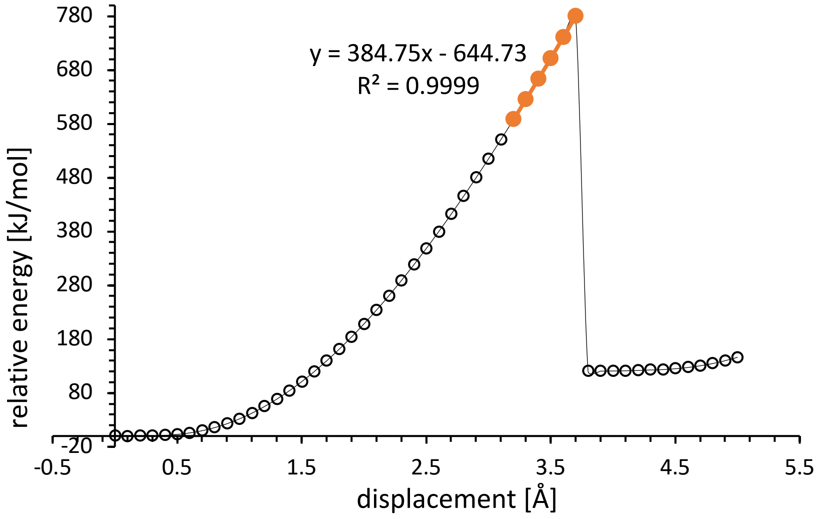	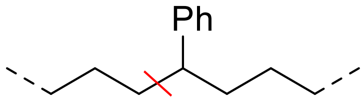<br>**9**<br>F_b = 6.50±0.01
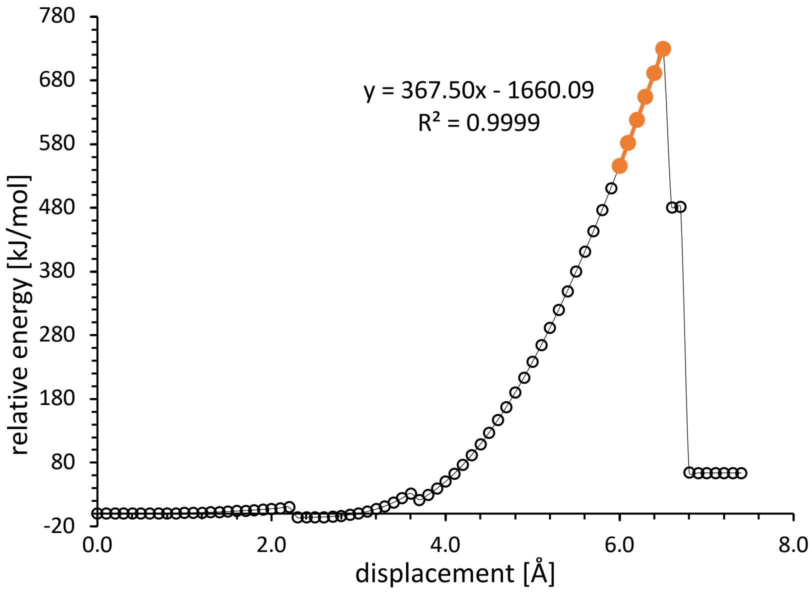	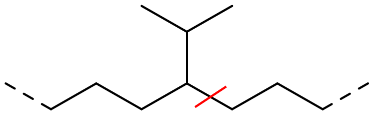<br>**10**<br>F_b = 6.10±0.03

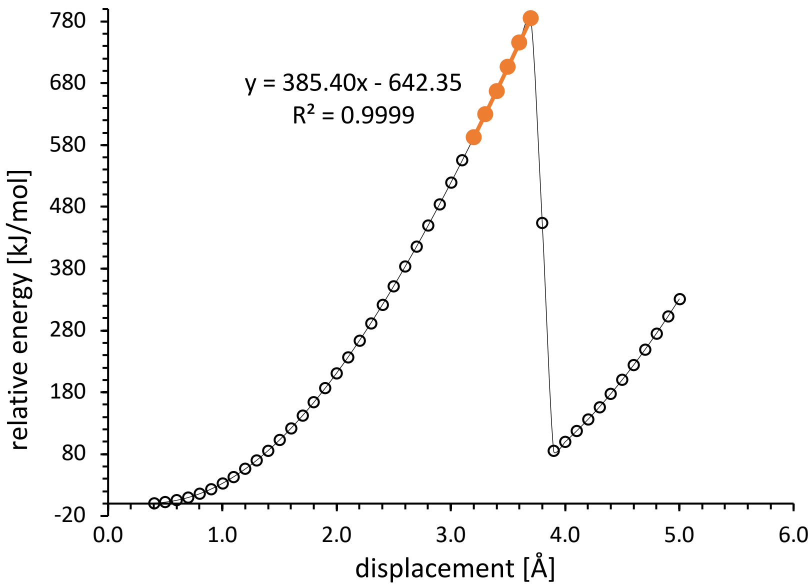

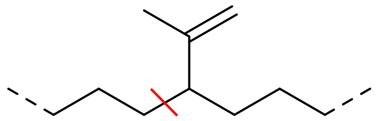

10'

F_b = 6.40±0.03

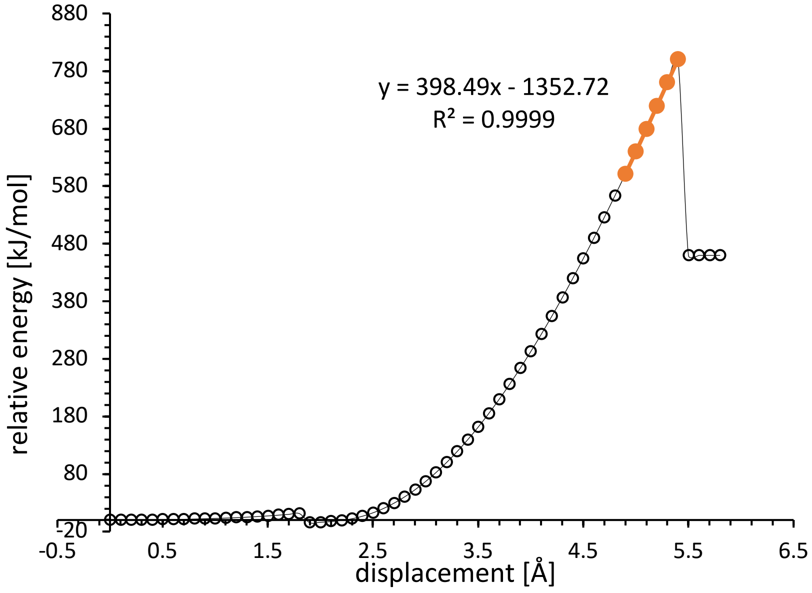

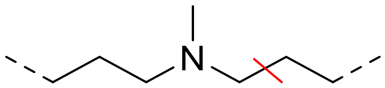

11

F_b = 6.62±0.03

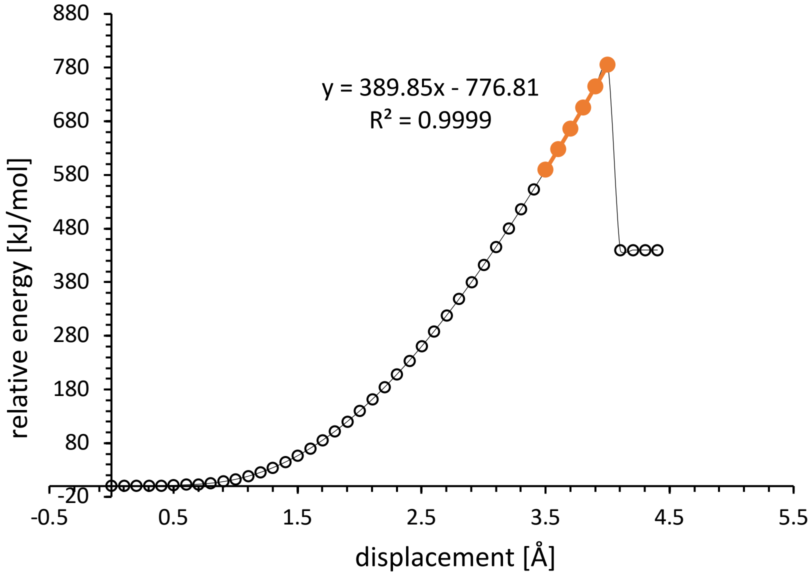

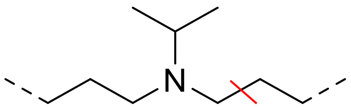

12

F_b = 6.48±0.03

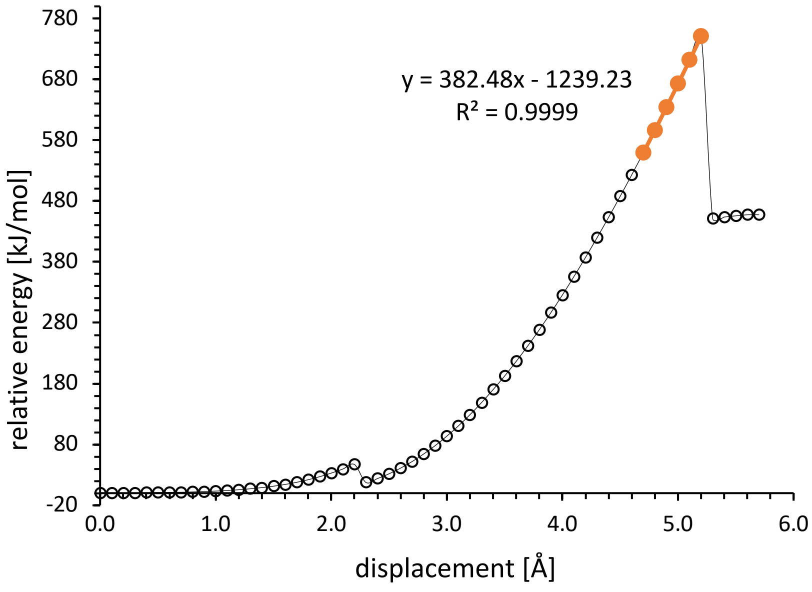

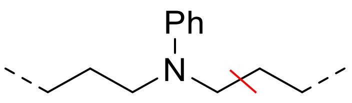

13

F_b = 6.35±0.04

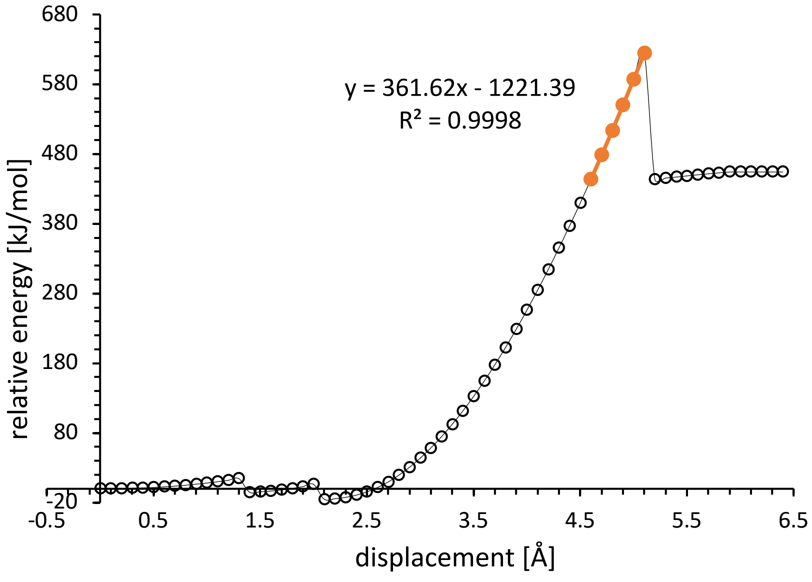

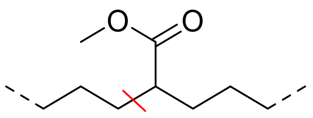

14

F_b = 6.01±0.04

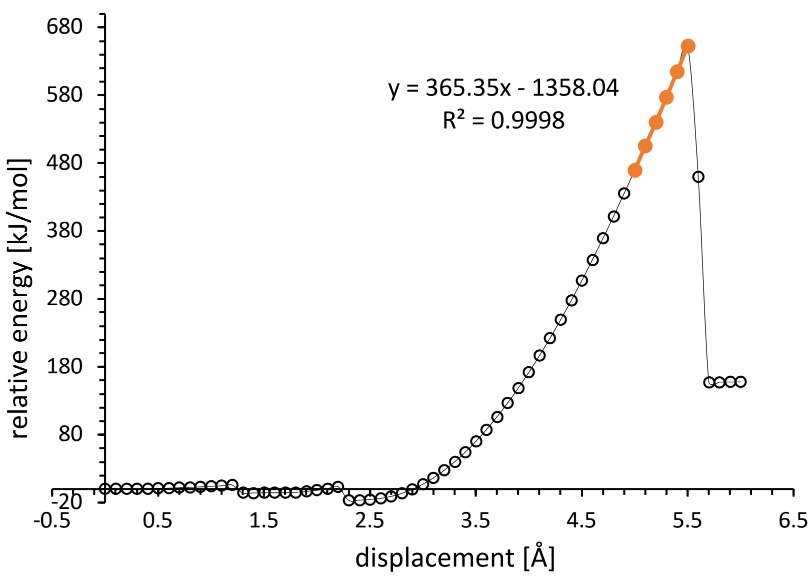

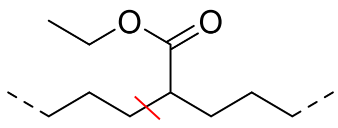

15

F_b = 6.07±0.05

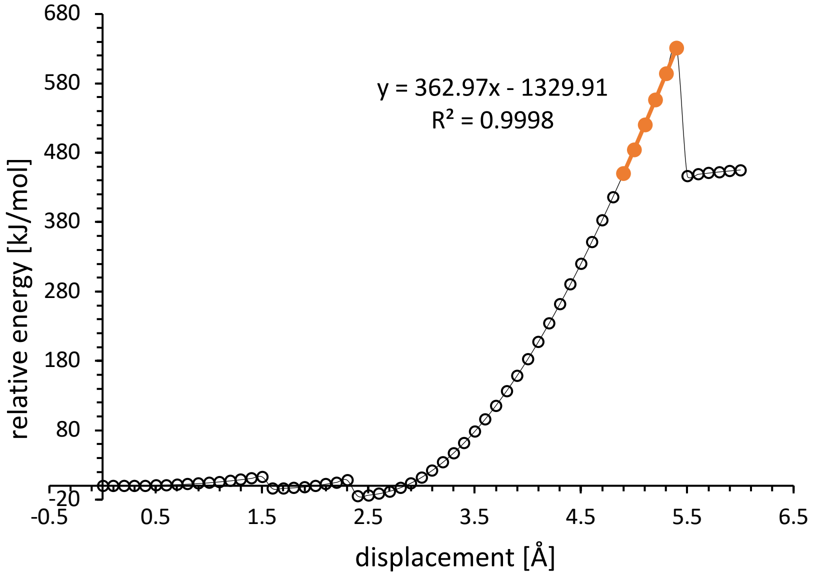

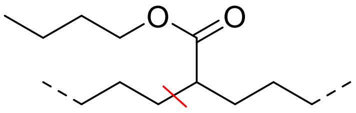

16

F_b = 6.03±0.04

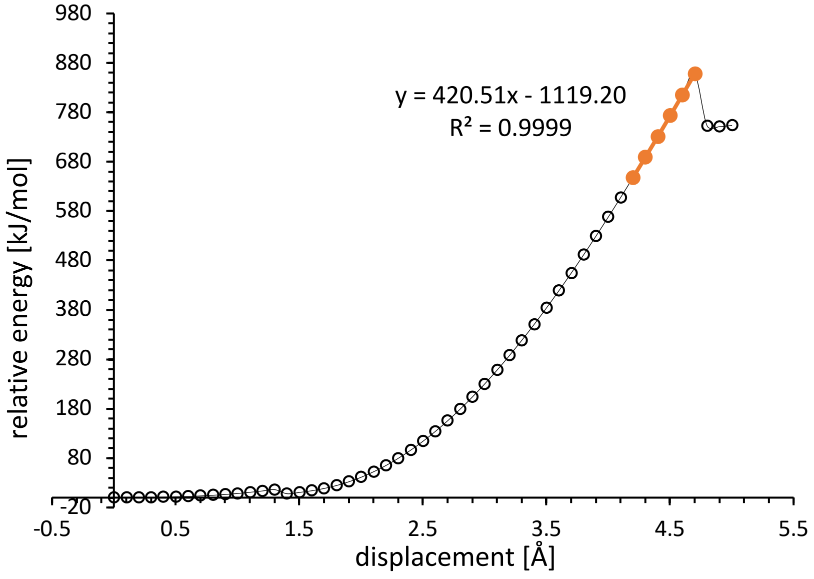

trans

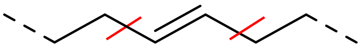

17

F_b = 7.02±0.03

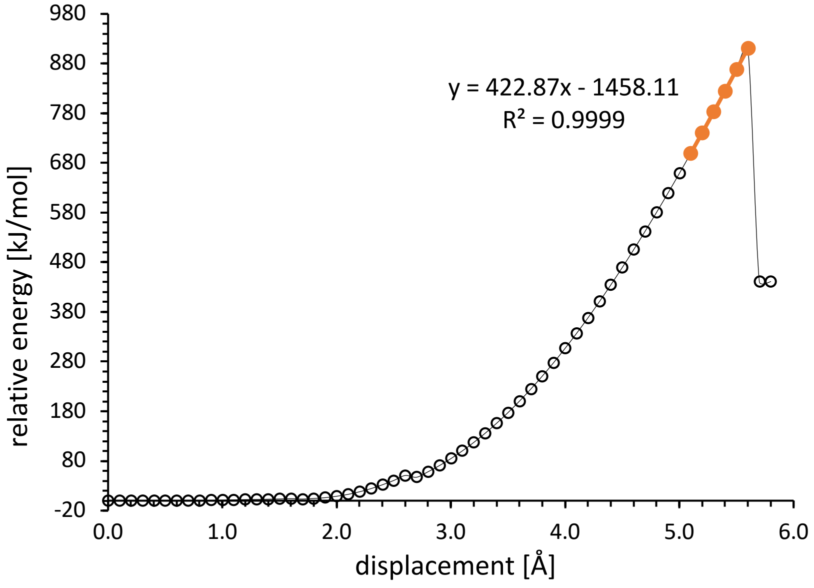

cis

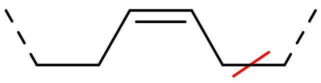

18

F_b = 7.02±0.03

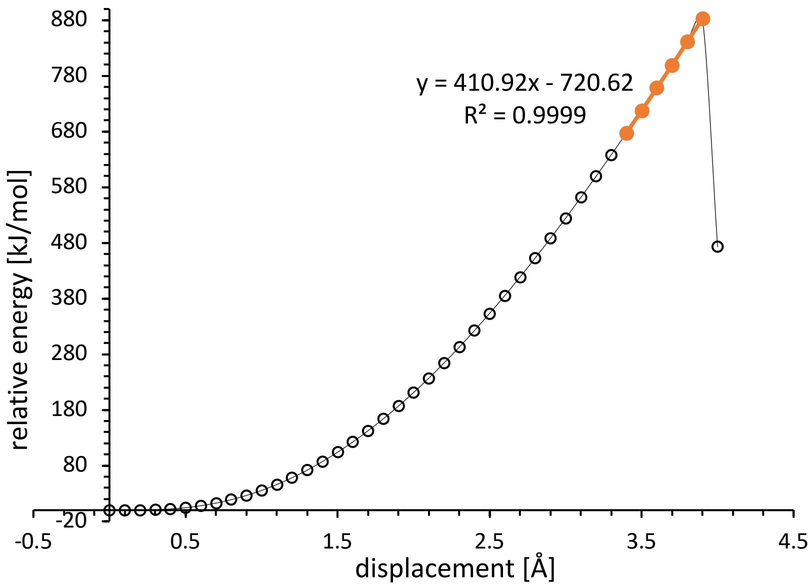	trans<br>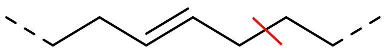<br>**19**<br>F_b = 6.83±0.03
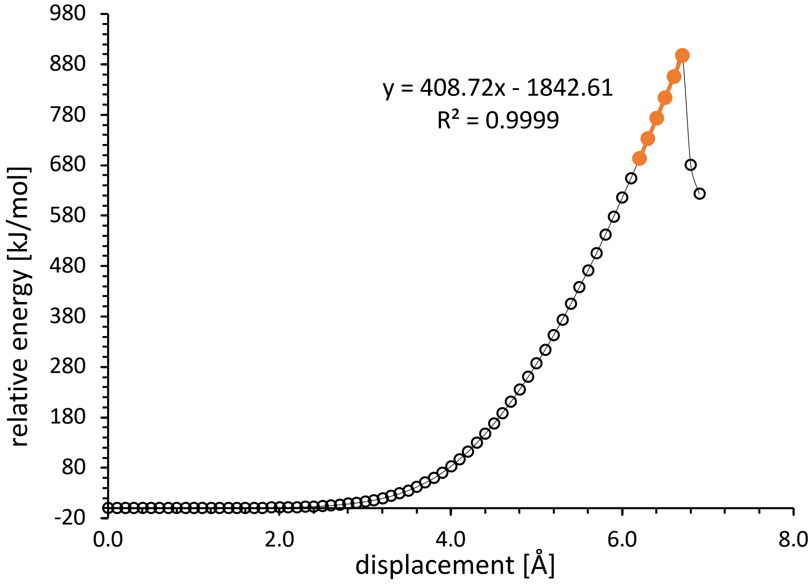	cis<br>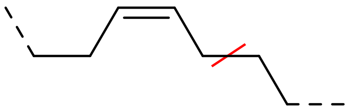<br>**20**<br>F_b = 6.79±0.03
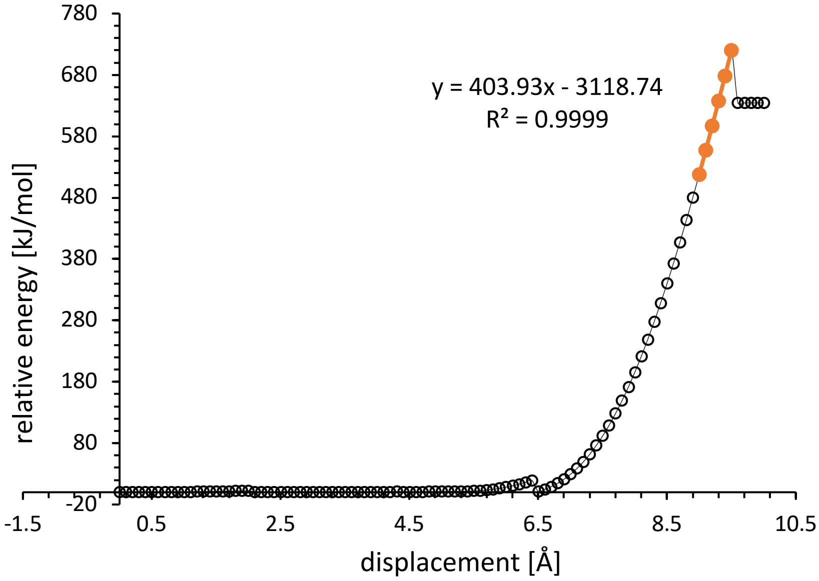	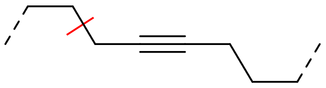<br>**21**<br>F_b = 6.71±0.04

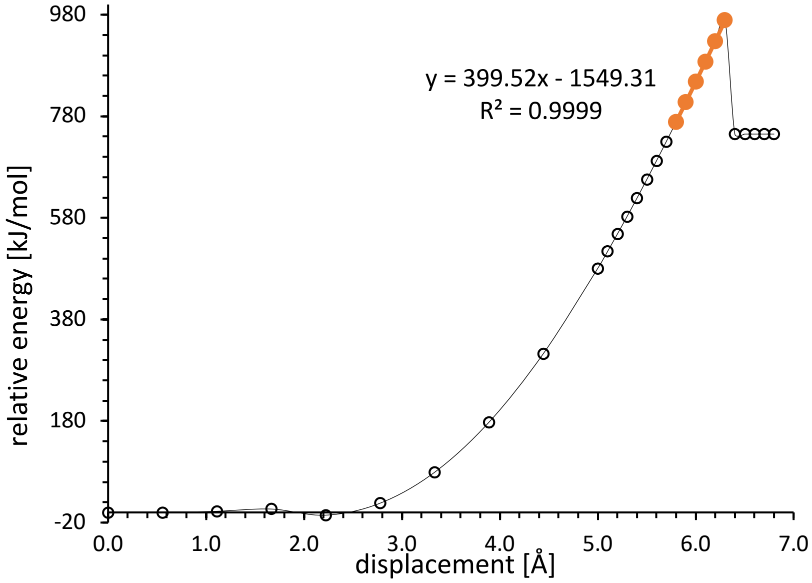	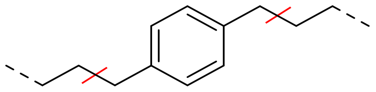<br>**22**<br>F_b = 6.64±0.03
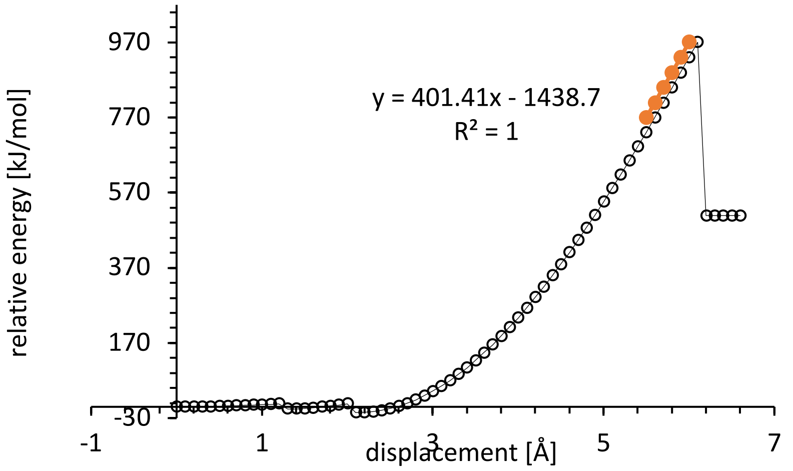	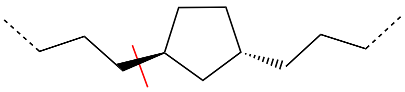<br>**23**<br>trans<br>F_b = 6.67±0.02
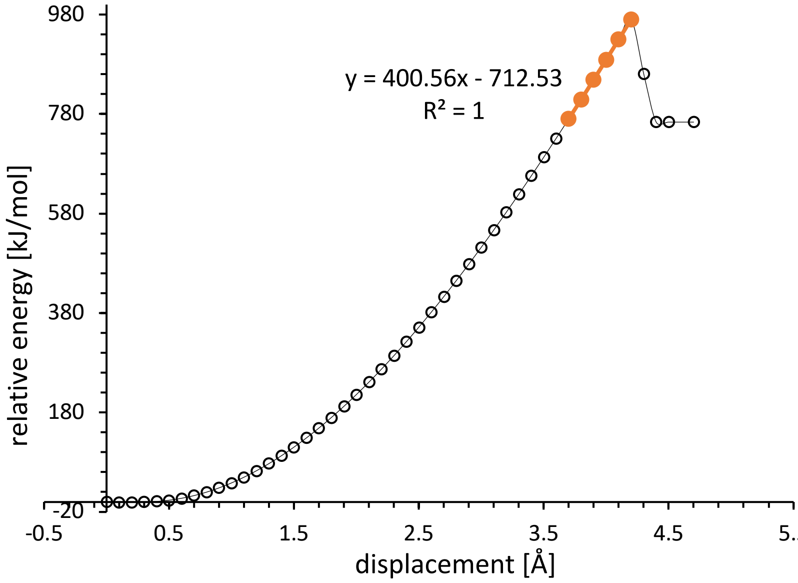	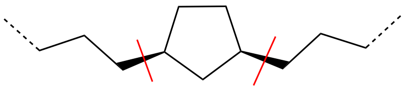<br>**24**<br>cis<br>F_b = 6.65±0.02

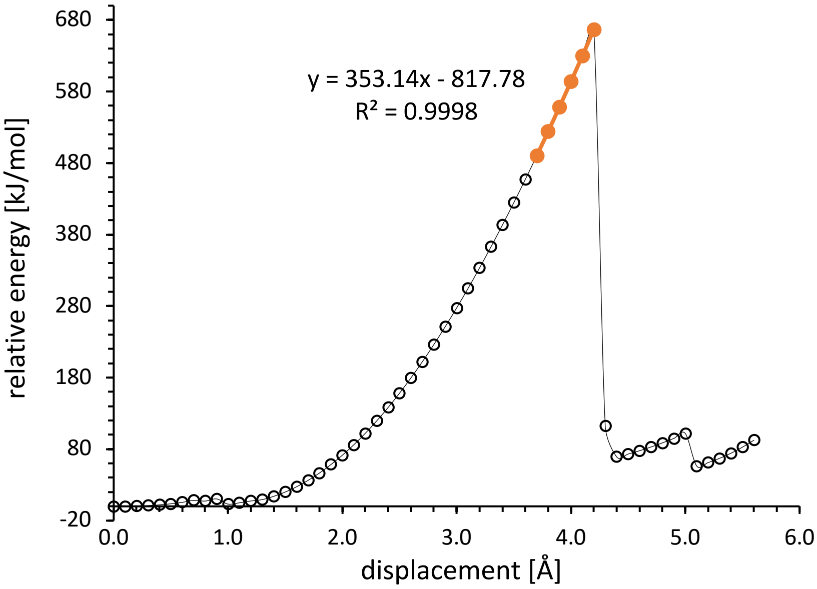

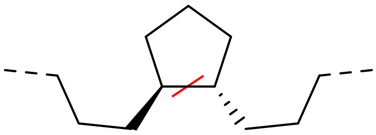

25

trans

F_b = 5.87±0.04

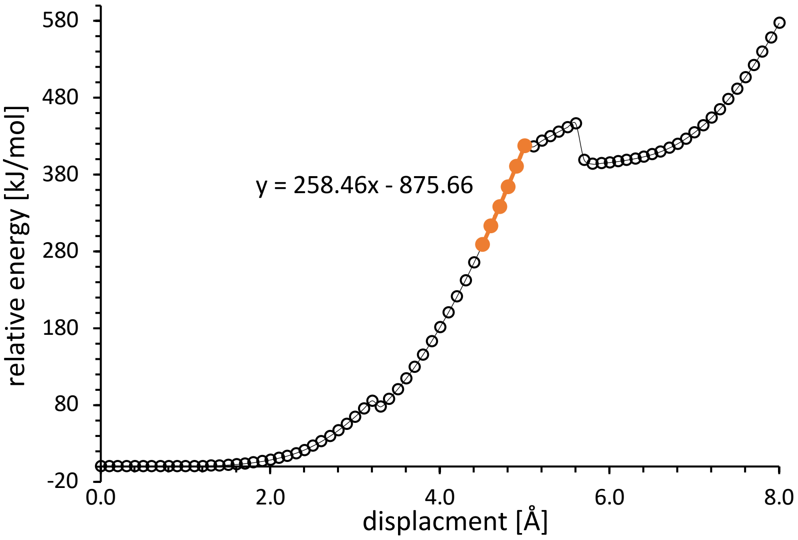

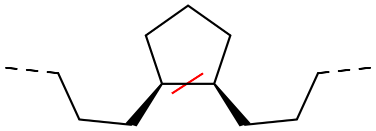

26

cis

F_b = 4.29±0.04

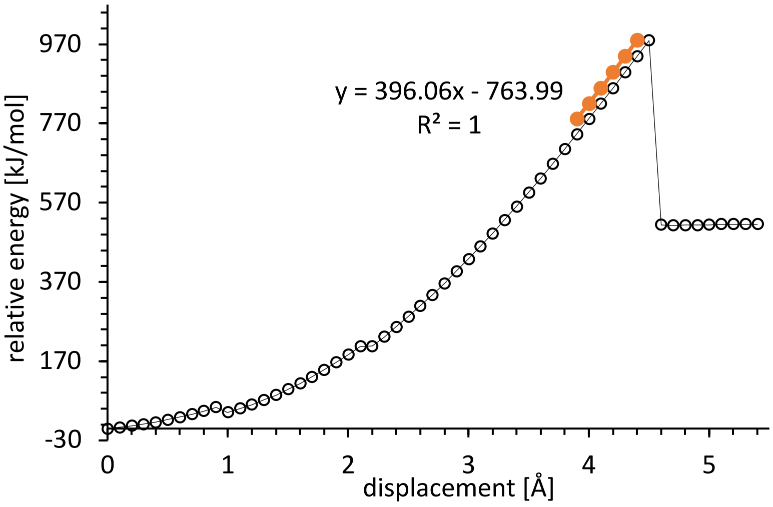

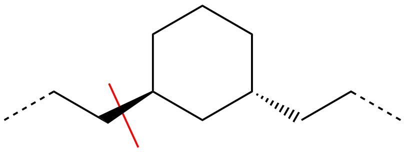

27

trans

Fb = 5.79±0.02

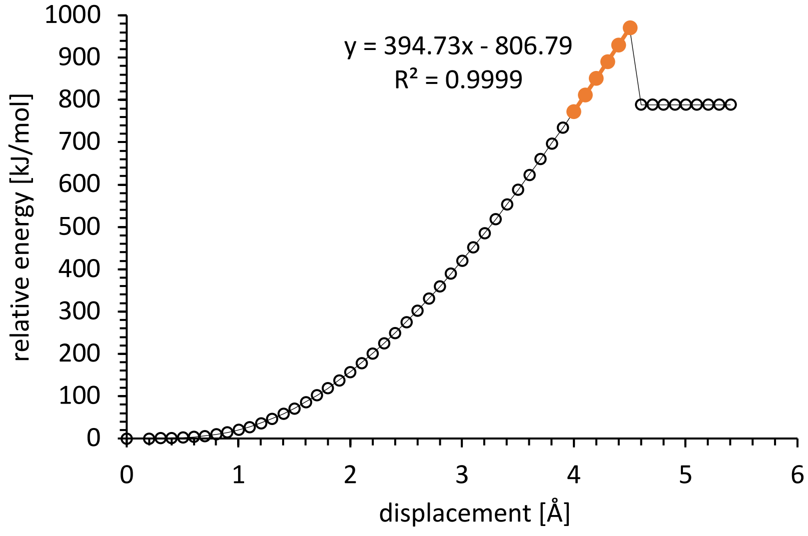	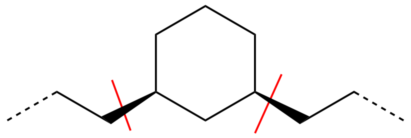<br>**28**<br>cis<br>Fb = 6.56±0.02
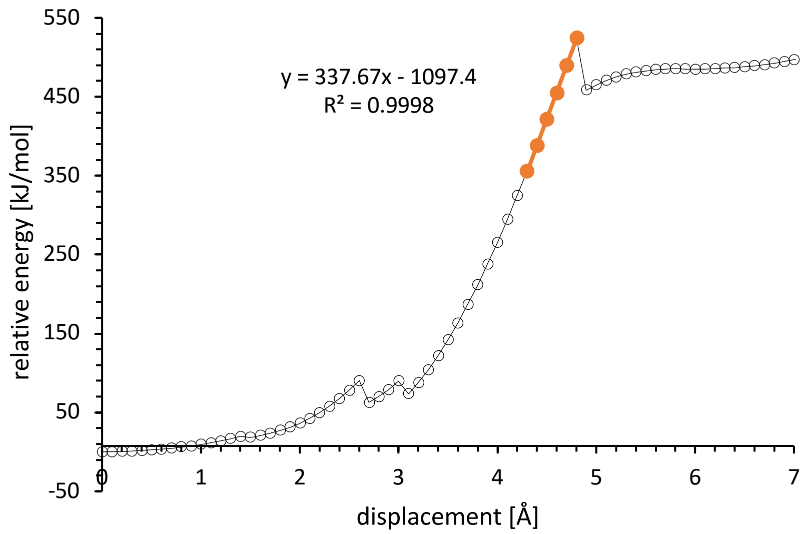	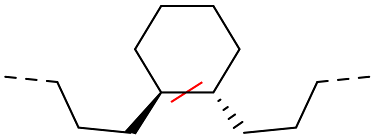<br>**29**<br>trans<br>F_b = 5.61±0.04
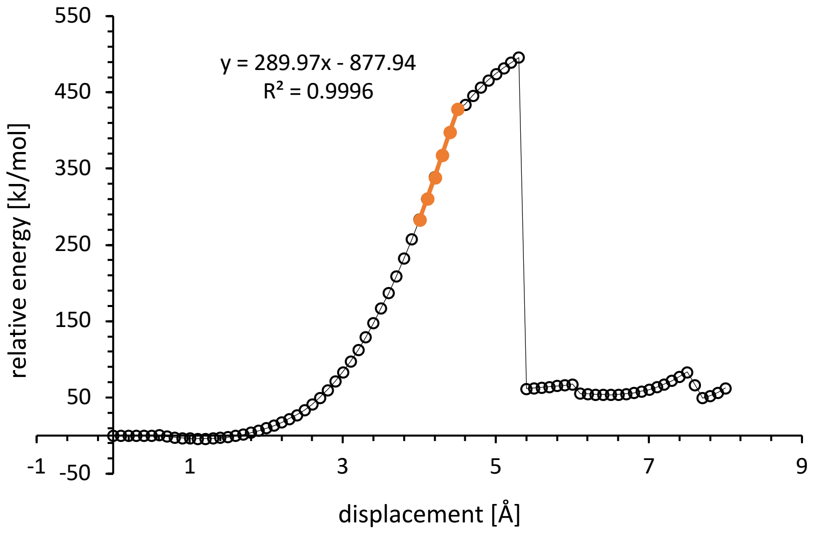	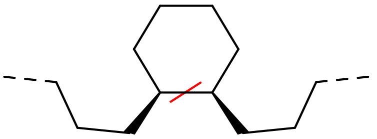<br>**30**<br>cis<br>F_b = 4.82±0.05

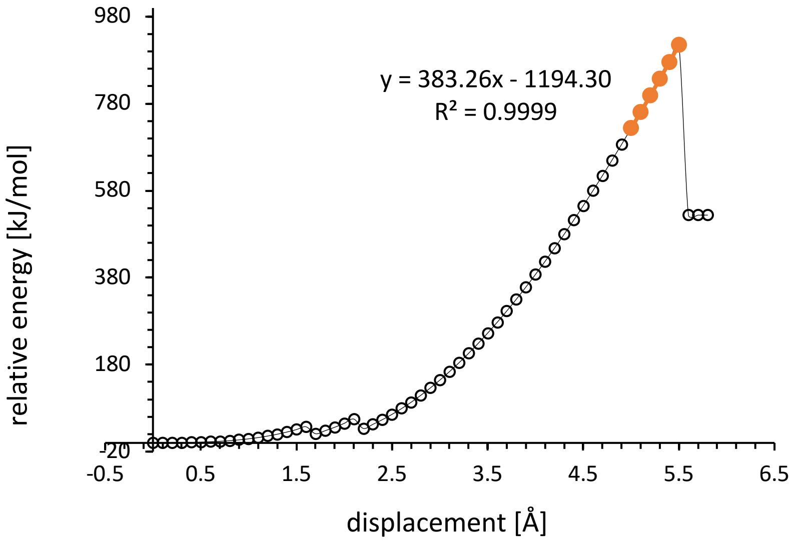

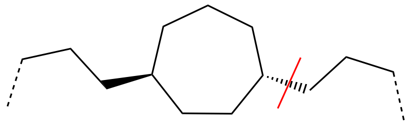

31

trans

$F_b = 6.37 \pm 0.02$

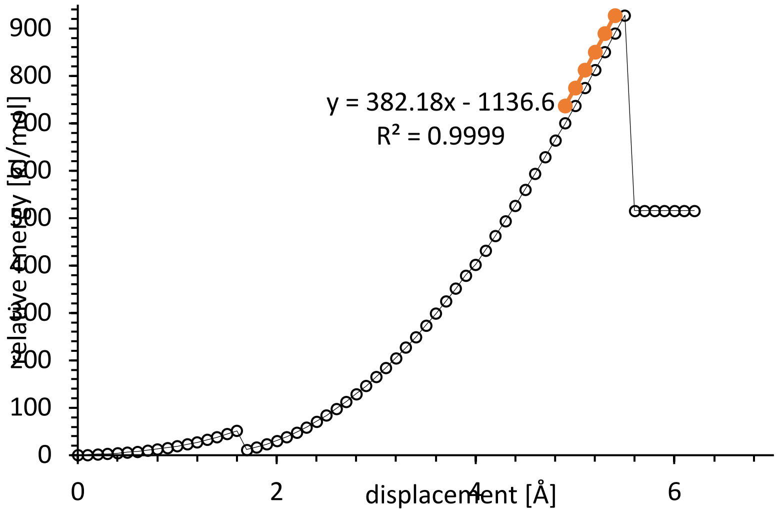

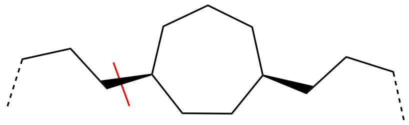

32

cis

$F_b = 6.35 \pm 0.03$

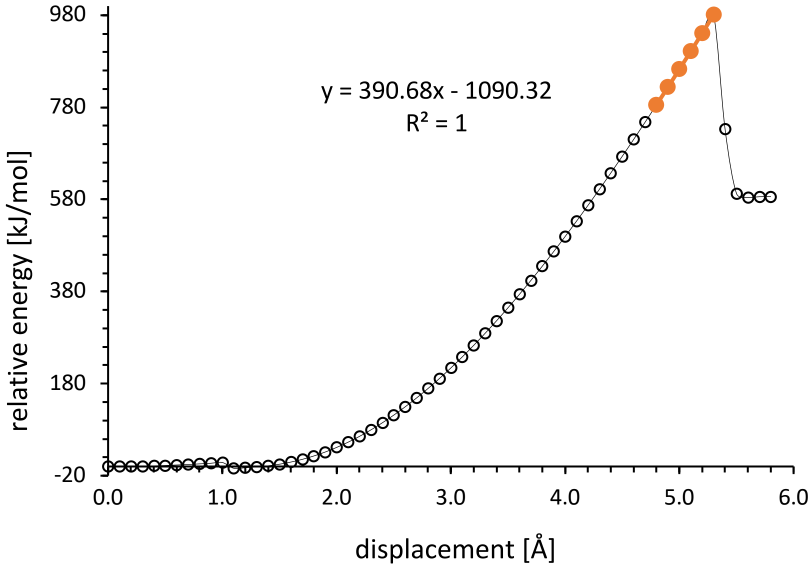

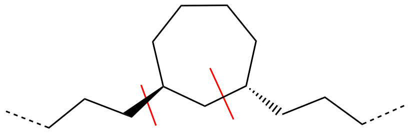

33

trans

$F_b = 6.49 \pm 0.02$

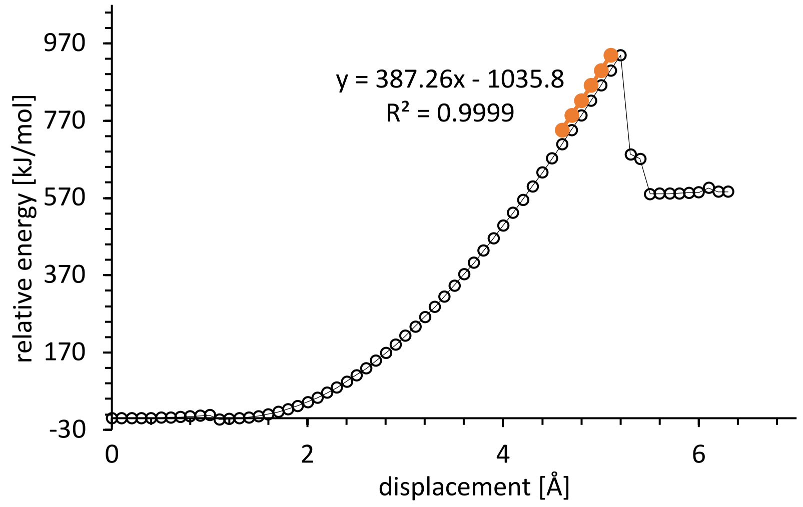	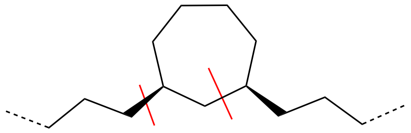<br>**34**<br>cis<br>F_b = 6.43±0.03
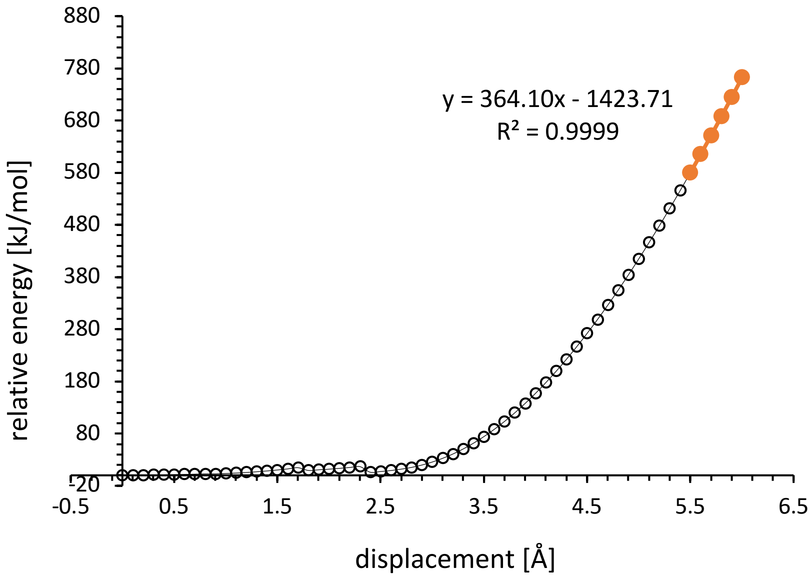	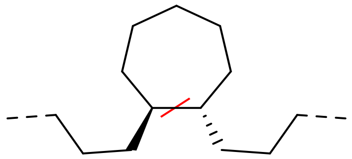<br>**35**<br>trans<br>F_b = 6.05±0.03
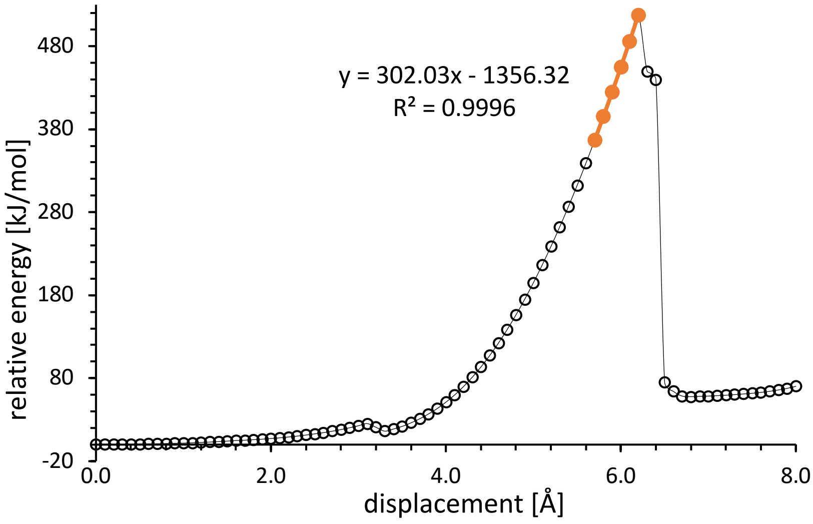	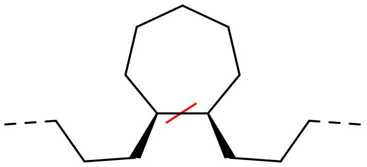<br>**36**<br>cis<br>F_b = 5.03±0.05

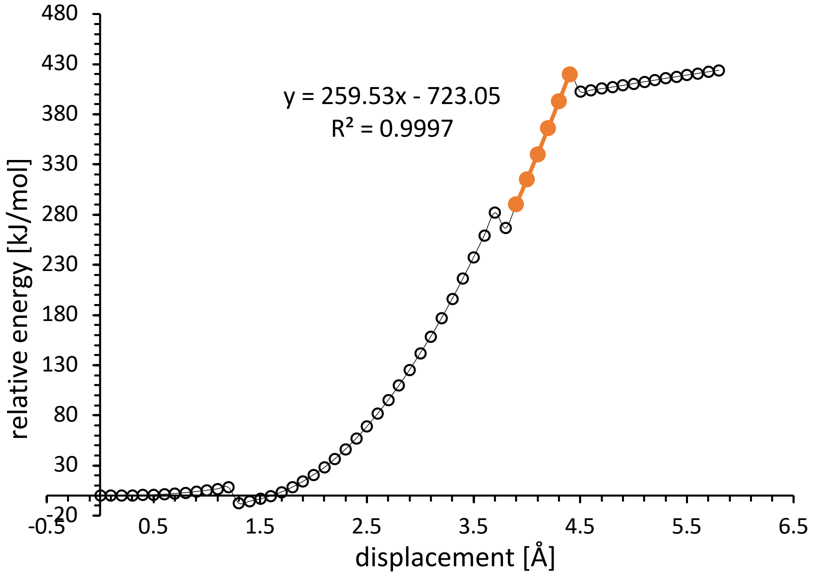

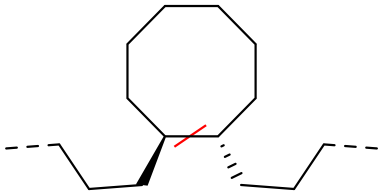

37

trans

F_b = 4.31±0.04

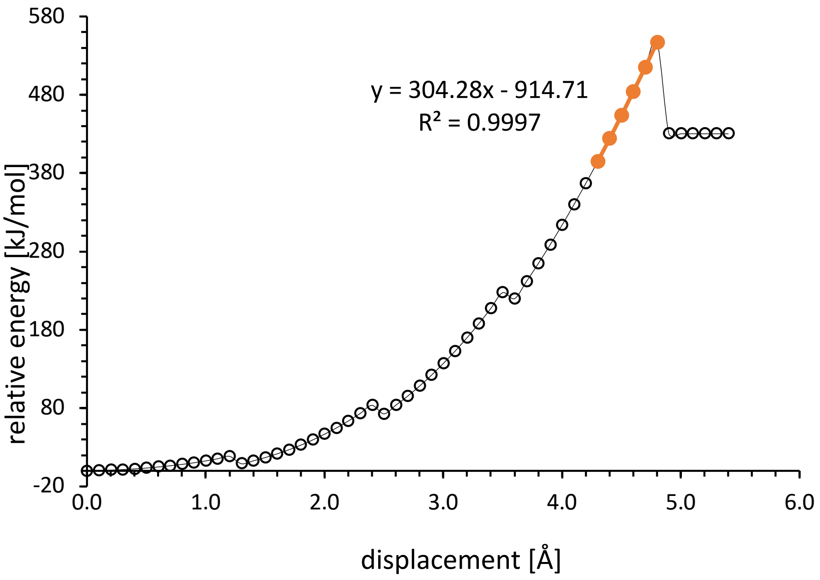

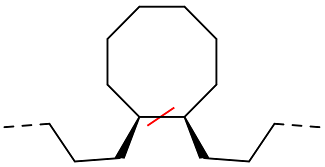

38

cis

F_b = 5.05±0.04

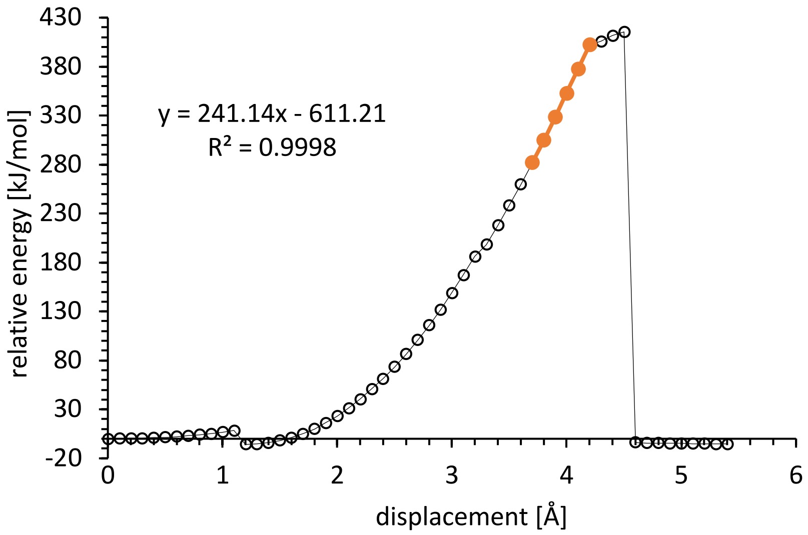

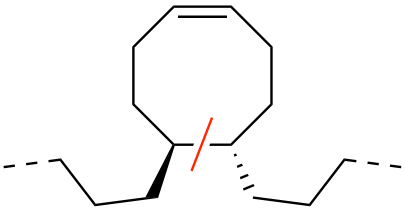

39

trans

F_b = 4.00±0.03

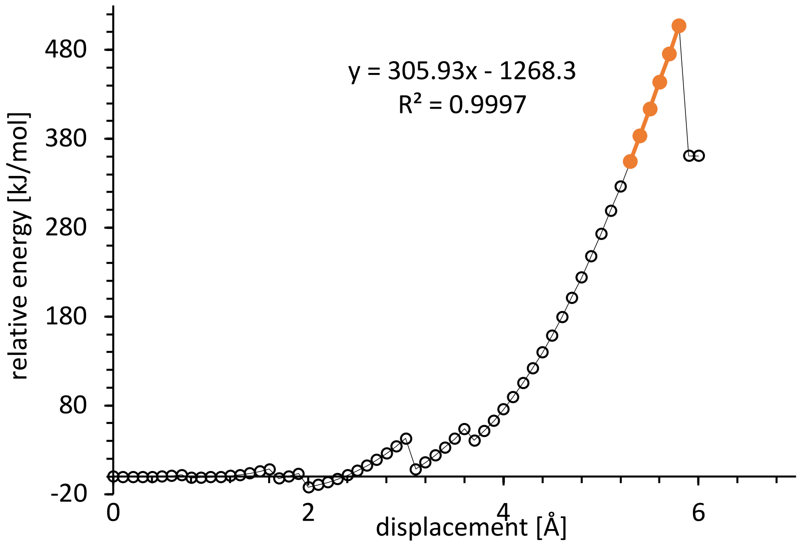

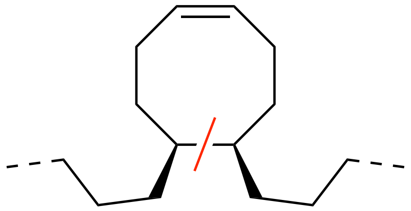

40

cis

F_b = 5.08±0.05

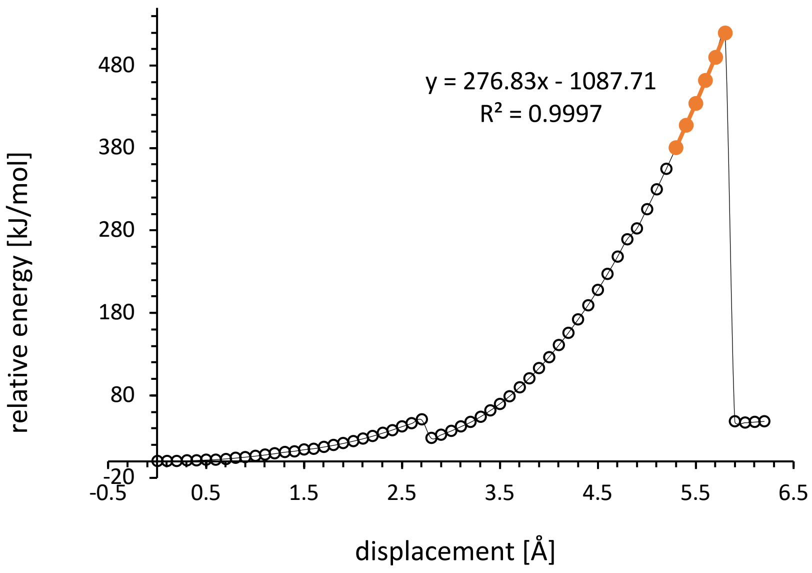

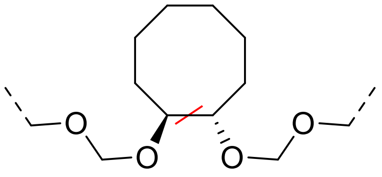

41

trans

F_b = 4.60±0.04

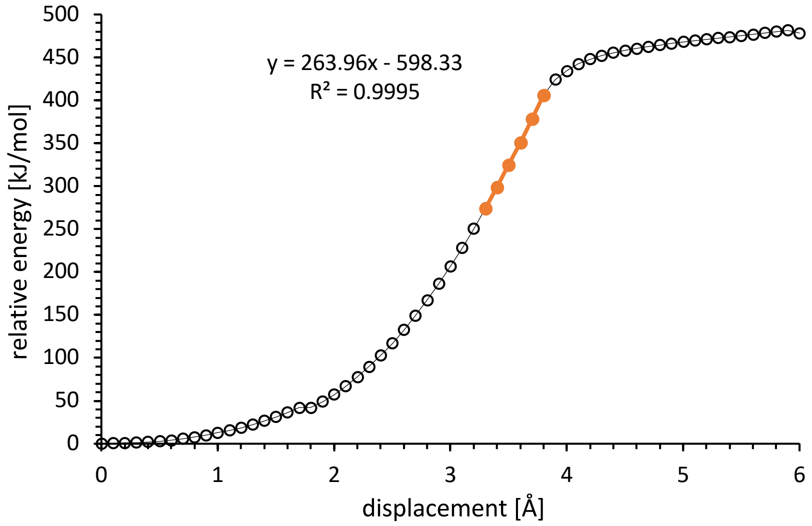

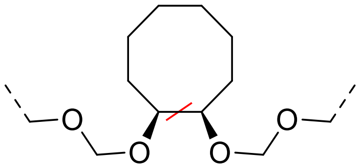

42

cis

F_b = 4.38±0.05

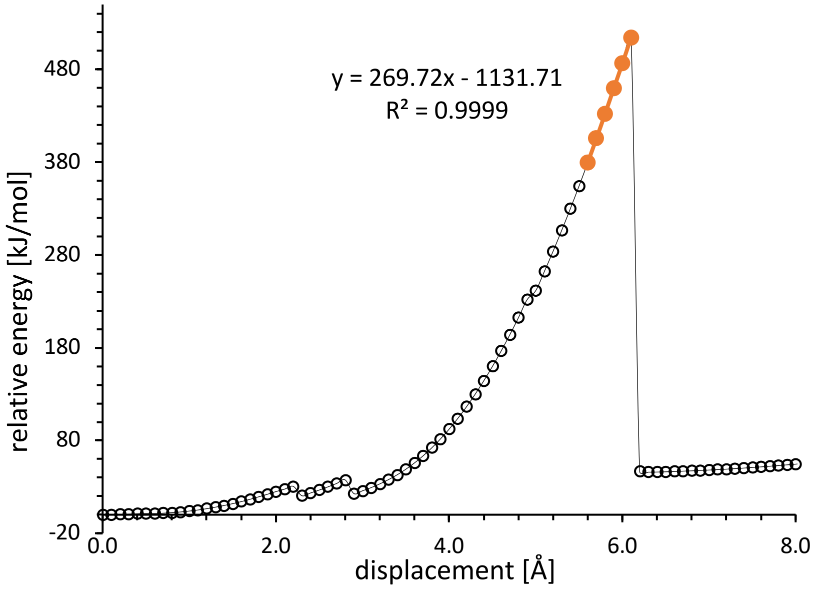 	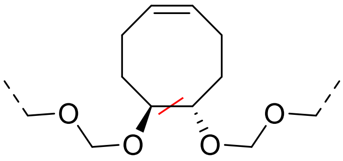<br>**43**<br>trans<br>F_b = 4.48±0.03
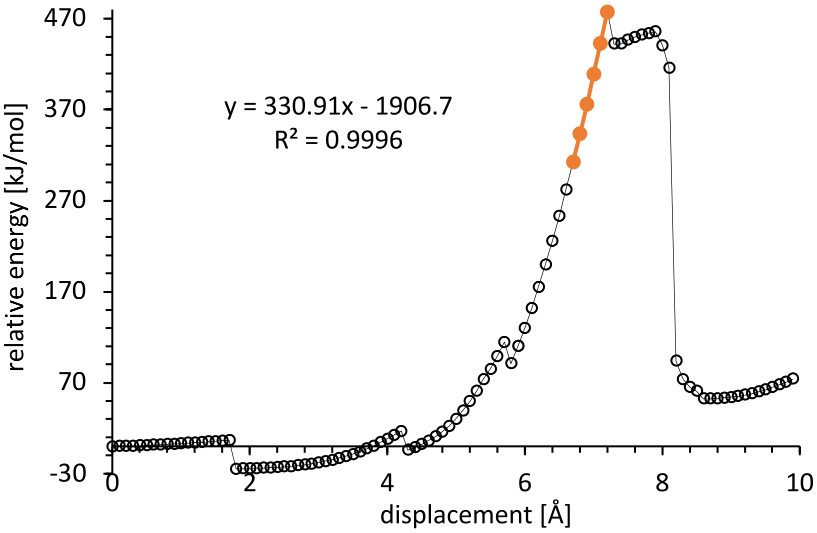 	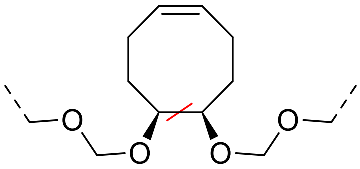<br>**44**<br>cis<br>F_b = 5.50±0.06
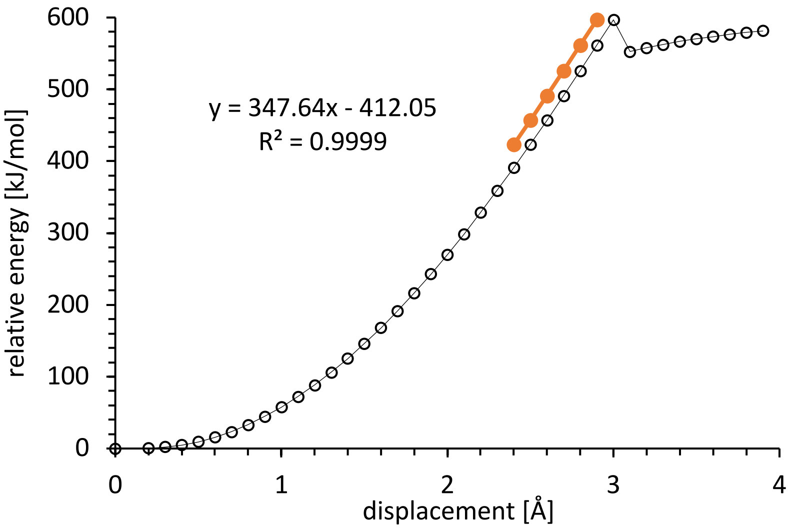 	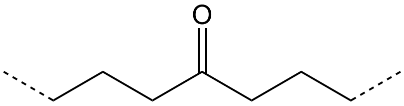<br>**45**<br>F_b = 5.77±0.03

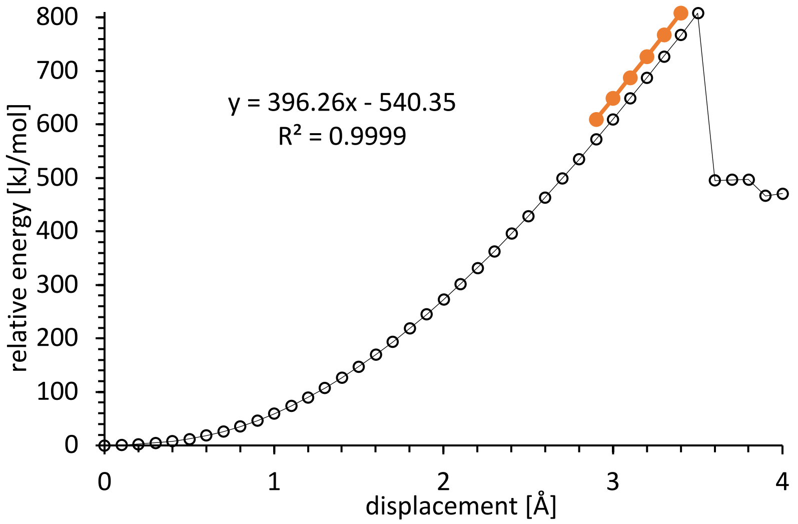

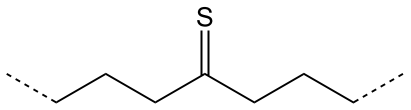

46

F_b = 6.58±0.03

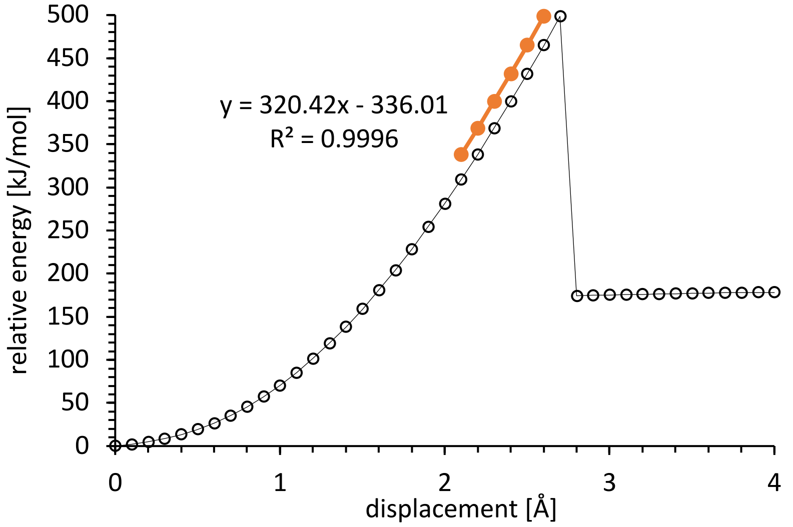

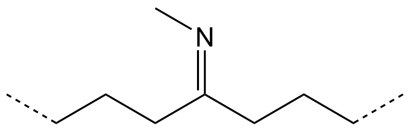

47

F_b = 5.32±0.05

Part III. Synthetic Procedures

Z-5-cyclooctene-*trans*-1,2-diol:

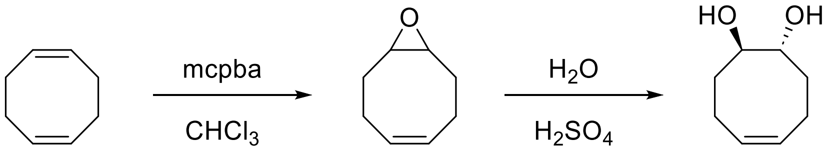

1,5-cyclooctadiene (1.08 gr, 10 mmol, 1.0 eq) was added to dry $CHCl_3$ (15 mL) and cooled to 0°C. mCPBA (1.39 gr, 8.05 mmol, 0.8 eq) in $CHCl_3$ (15 mL) was added dropwise during 1 h. The mixture was further stirred at room temperature for 12 h. The reaction was then quenched with saturated $Na_2S_2O_3$ solution (30 mL). The $CHCl_3$ phase was collected and washed with Na_2CO_3 (2 x 30 mL) and brine (3 x 30 mL). The extract was dried over Na_2SO_4 and evaporated under vacuum. The product was added to water (20 mL), two drops of H_2SO_4 were added and the mixture was stirred for 48 h. The product was extracted with DCM, the organic phases combined, washed with water, dried over Na_2SO_4, and evaporated. (Yellowish oil product, 0.9 g, 80% yield), ^{1}H NMR (400 MHz, $CDCl_3$) δ 5.60 (dd, J = 7.4, 2.9 Hz, 1H), 3.71 – 3.63 (m, 1H), 2.44 (s, 1H), 2.39 – 2.30 (m, 1H), 2.20 – 2.04 (m, 3H), 1.65 – 1.54 (m, 1H).^{13}C NMR (101 MHz, $CDCl_3$) δ 129.3, 74, 33.5, 22.8. (according to reported spectra)[40]

Polyphthalaldehyde (PPA) dianionic polymerization – general procedure:

Monomer *ortho*-phthalaldehyde (oPA) was recrystallized 3 times from DCM / hexane (5:2 solution, dried under vacuum for 24 h and stored in a glovebox freezer. Inside the glove box, trice recrystallized oPA (1 gr, 7.45 mmol, 1.0 eq) was added to a Schlenk flask, followed by anhydrous THF (12.4 mL) and a solution of an initiator in THF (see table below). The flask was sealed and removed from the glove box. The flask was connected to a Schlenk nitrogen line and cooled to -78°C. After ~10 min, 1-tert-Butyl-2,2,4,4,4-pentakis(dimethylamino)-2Λ^5,4Λ^5-catenadi (phosphazene) in THF solution (P_2tBu, ~2.0 mol/L, 0.054 mmol, 28 µl) was added into the flask via a septum. After mixing for 4 h at -78°C, pyridine (30 µL, 0.37 mmol) was added, followed by phenyl isocyanate (74 µL, 0.34 mmol). After 3 h, the solution was allowed to return to room temperature and the flask content was precipitated in methanol. The obtained polymer was filtered and dried under vacuum for 48 h and stored under argon in a freezer. NMR of PPA-b obtained according to previously reported in literature.[63] ^{1}H NMR (300 MHz, CD_2Cl_2) δ 7.86 – 7.18 (m, 4H), 7.18 – 6.32 (m, 2H). (Figure S3).

Polymer	initiator	n [mmol]
PPA-a	*Z*-5cyclooctene-1,2-diol	0.007 mmol, 0.1 mol%
PPA-b	1,7-heptanediol	0.007 mmol, 0.1 mol%
PPA-c	1-heptanol	0.014 mmol, 0.2 mol%

Part IV. NMR Spectra

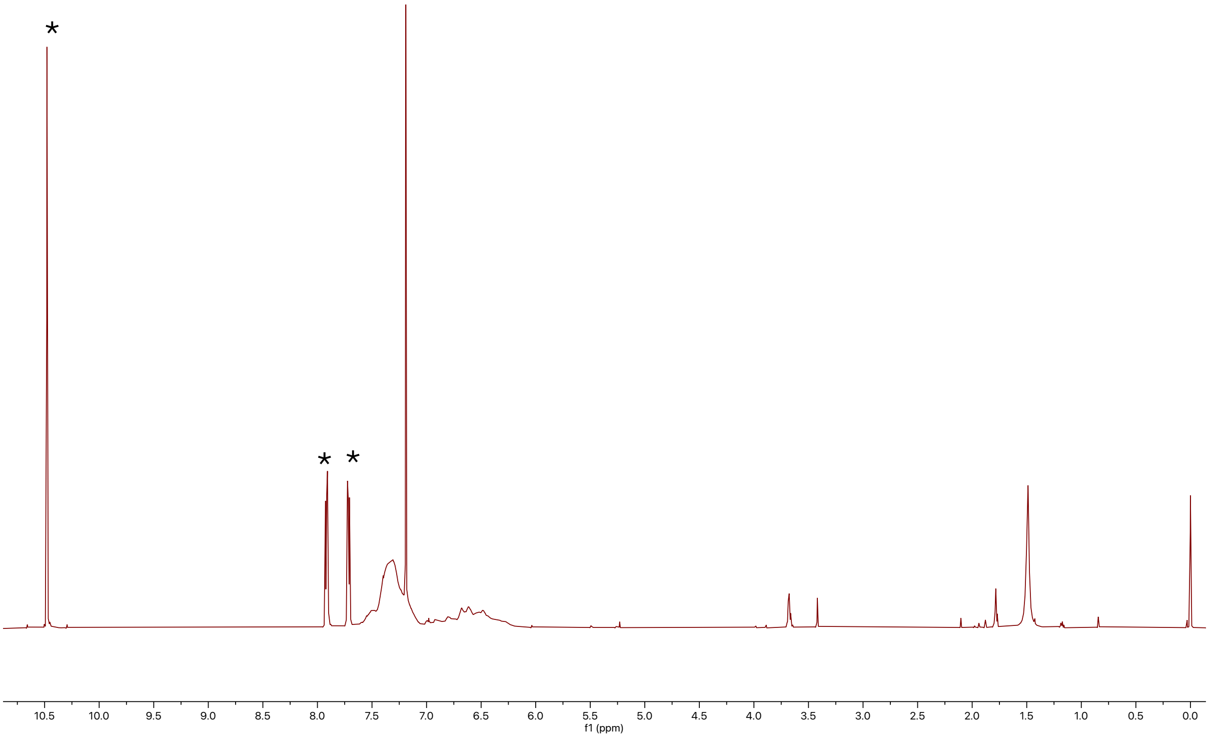

Figure S2. ^{1}H-NMR and ^{13}C-NMR of PPA-a (CD_3Cl), *peaks of trace monomer

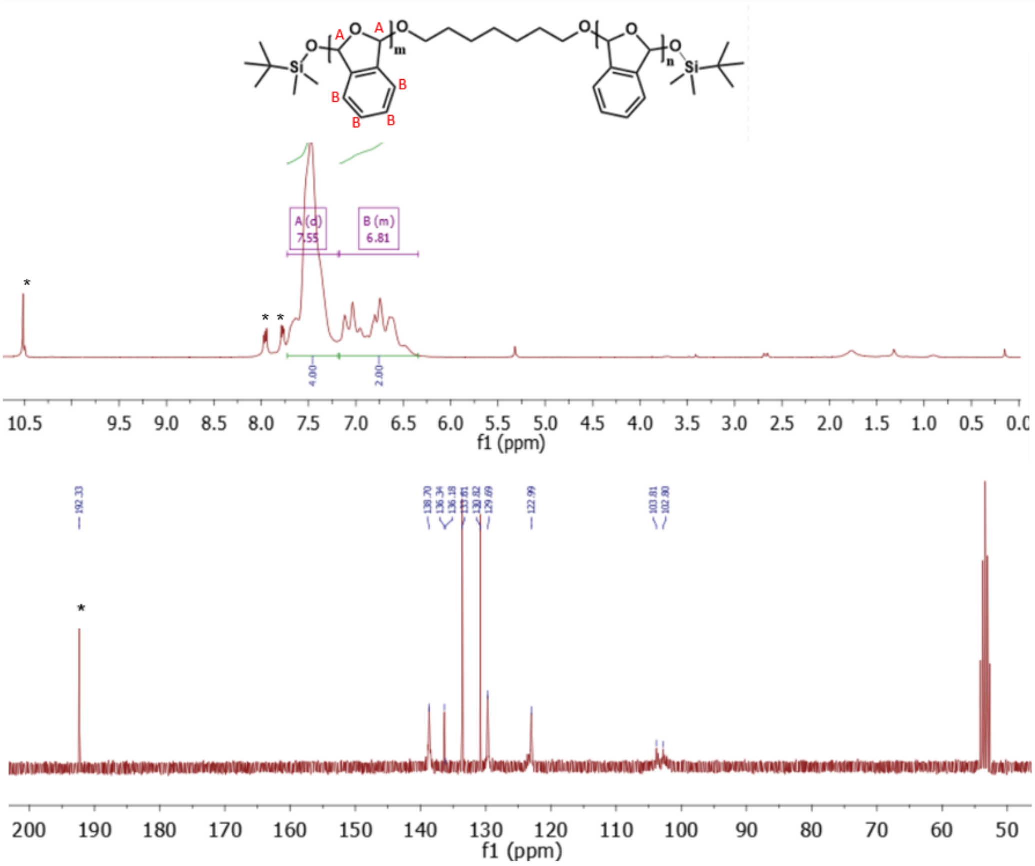

Figure S3. ^{1}H-NMR and ^{13}C-NMR of PPA-b (CD_2Cl_2), *peaks of trace monomer

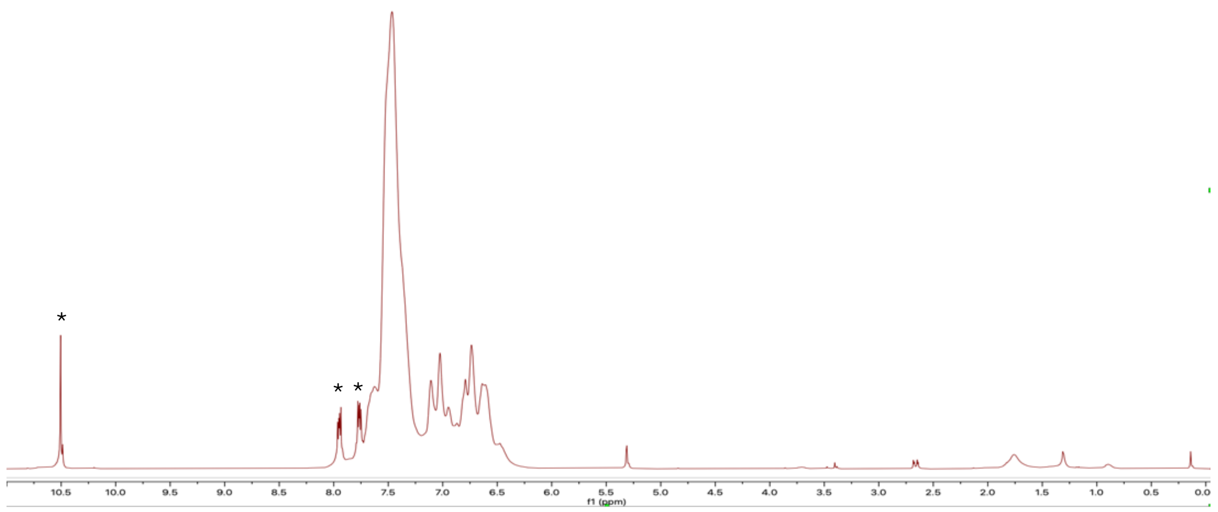

Figure S4. ^{1}H-NMR of PPA-c (CD_2Cl_2), *peaks of trace monomer

Part V. GPC Analysis

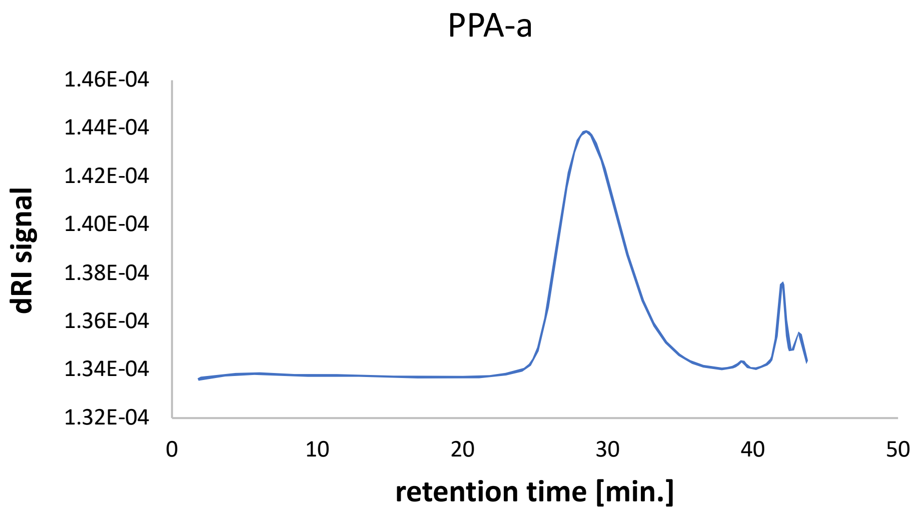

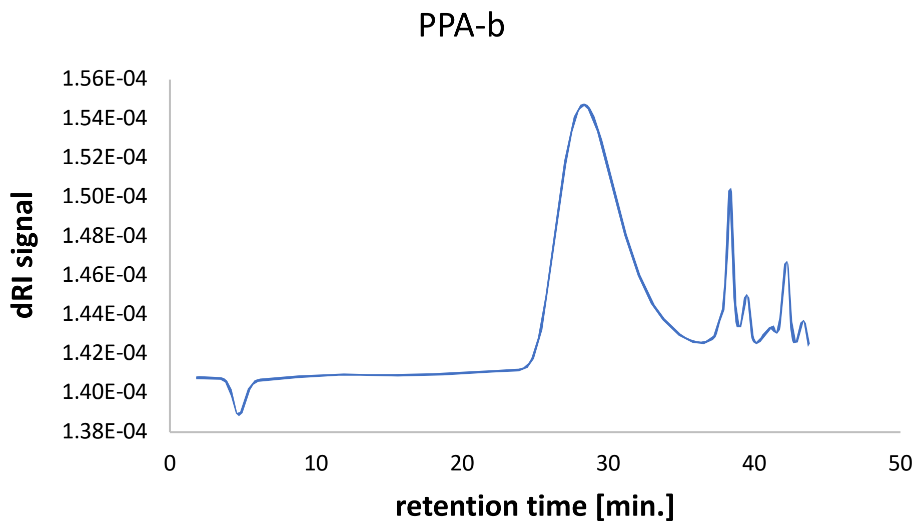

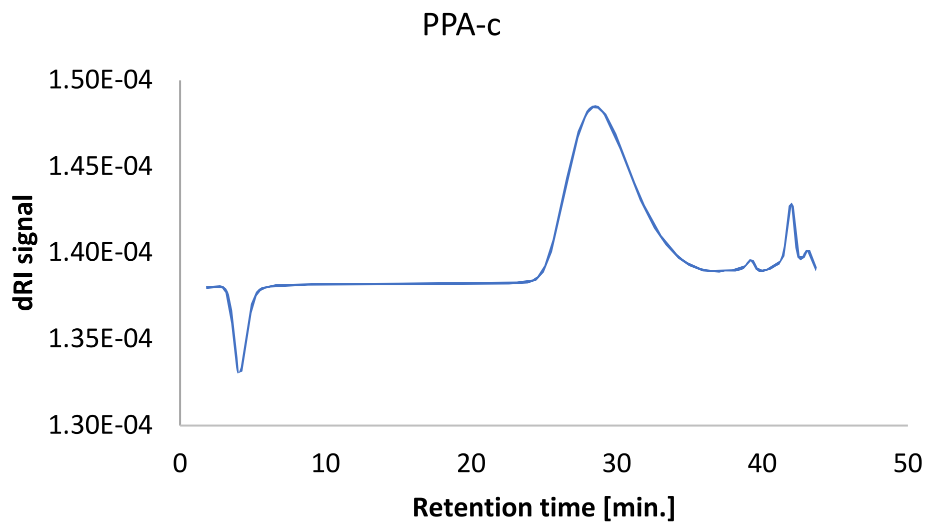

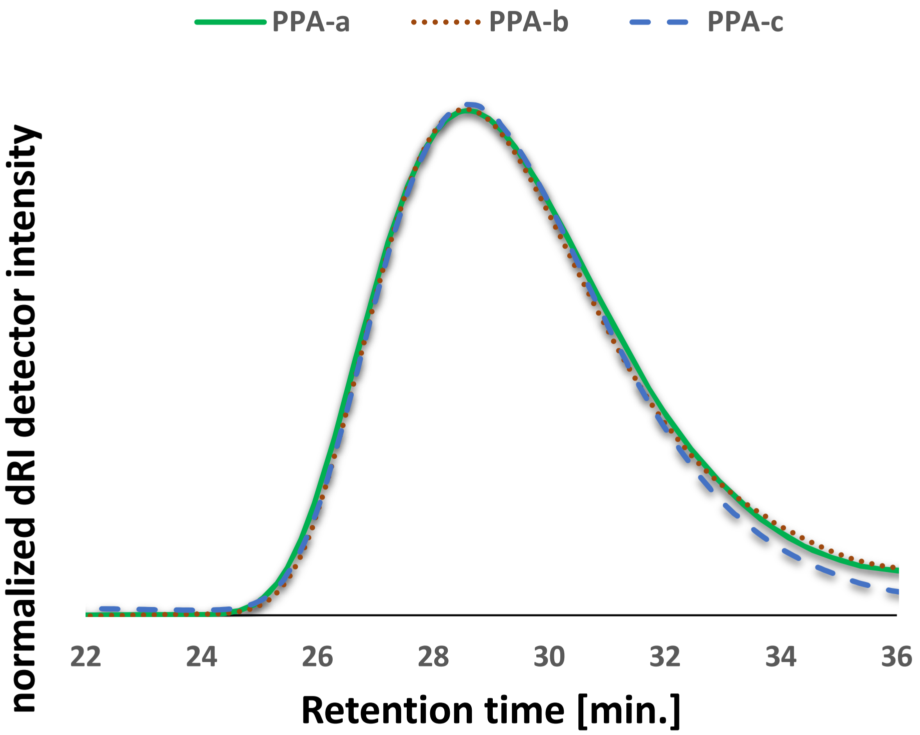

Figure S5. Zoom in the polymer peak of GPC of PPA-(a-c). GPC run as described above, in THF, 1 ml/min.

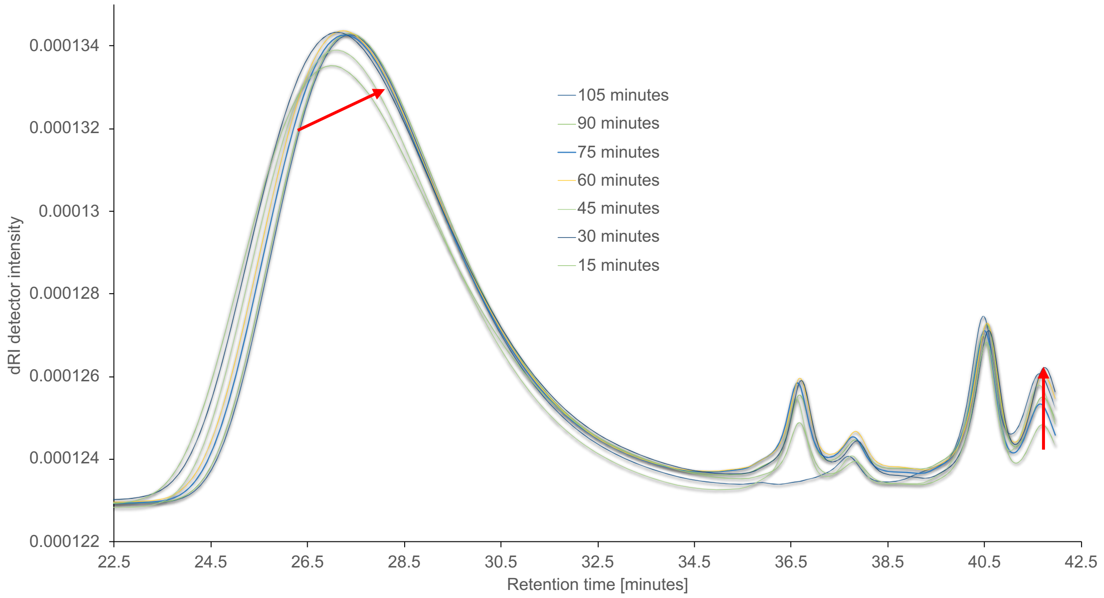

Figure S6. PPA-a GPC dRI spectrum change over sonication time (1 of 3 independent runs). GPC run as described above, in THF, 1 ml/min. Polymer peak area decreases while slightly moving to the right, monomer (low Mw peak) increases with time.

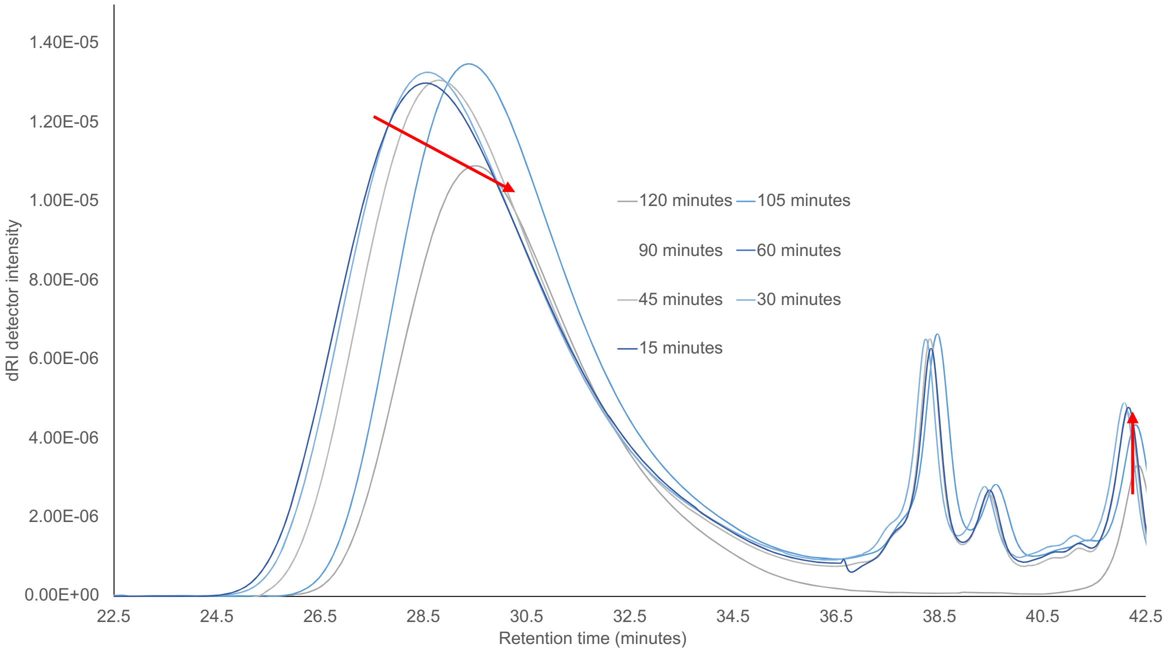

Figure S7. PPA-b GPC dRI spectrum change over sonication time (1 of 3 independent runs). GPC run as described above, in THF, 1 ml/min. Polymer peak area decreases while moving to the right, monomer (low Mw peak) increases with time.

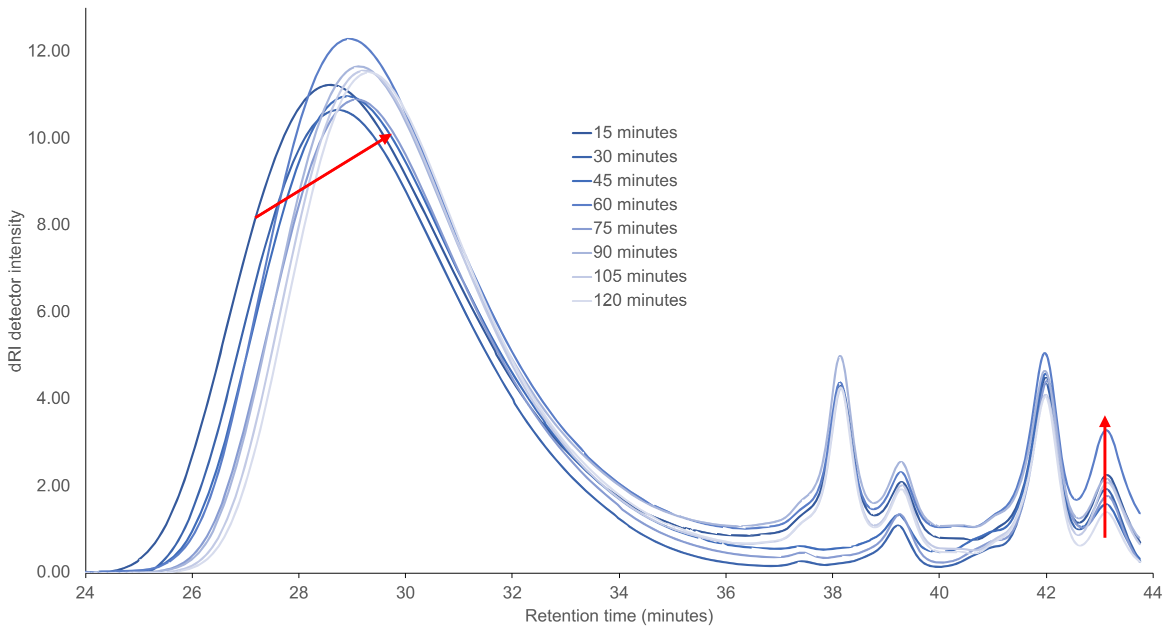

Figure S8. PPA-c GPC dRI spectrum change over sonication time (1 of 3 independent runs). GPC run as described above, in THF, 1 ml/min. Polymer peak area decreases while moving to the right, monomer (low Mw peak) increases with time.

Part VI. Statistical Analysis

Statistical analysis

Statistical analysis was performed using student's t-test with a minimum confidence level of 0.1 for statistical significance and assuming unequal variables. Calculation was performed using Microsoft Excel data analysis algorithm.

Table S2: Student t test results comparing CoGEF different families described in Figure 20. All results show statistically significant difference between each family pair.

	Tertiary	Quaternary	Tertiary with C=O	Cis cyclooctane/ene	Trans cyclooctane/ene
Secondary	3.446	7.223	12.460	12.013	22.825
Tertiary		3.063	8.371	10.235	20.700
Quaternary			8.775	9.460	20.960
Tertiary with C=O				7.343	18.303
Cis cyclooctane/ene					5.020
Trans cyclooctane/ene					

Table S3: Student t test results comparing depolymerization rates of every experiment pair in differently initiated PPA described in Figure 18. Red color indicates no statistical difference between the pair.

	PPA-b initial depolymerization rate	PPA-c initial depolymerization rate
PPA-a initial depolymerization rate	7.018	2.297
PPA-b initial depolymerization rate		0.982
PPA-c initial depolymerization rate		
